# Genetic regulation of gene expression in the epileptic human hippocampus

**DOI:** 10.1093/hmg/ddx061

**Published:** 2017-03-03

**Authors:** Nasir Mirza, Richard Appleton, Sasha Burn, Daniel du Plessis, Roderick Duncan, Jibril Osman Farah, Bjarke Feenstra, Anders Hviid, Vivek Josan, Rajiv Mohanraj, Arif Shukralla, Graeme J. Sills, Anthony G. Marson, Munir Pirmohamed

**Affiliations:** 1 Department of Molecular & Clinical Pharmacology, University of Liverpool, Liverpool L69 3GL, UK; 2 The Roald Dahl EEG Unit, Paediatric Neurosciences Foundation, Alder Hey Children's NHS Foundation Trust, Liverpool L12 2AP, UK; 3 Department of Neurosurgery, Alder Hey Children's NHS Foundation Trust, Liverpool L12 2AP, UK; 4 Department of Cellular Pathology, Salford Royal NHS Foundation Trust, Salford M6 8HD, UK; 5 Department of Neurology, Christchurch Hospital, Christchurch 8140, New Zealand; 6 Department of Neurosurgery, The Walton Centre NHS Foundation Trust, Liverpool L9 7LJ, UK; 7 Department of Epidemiology Research, Statens Serum Institut, Copenhagen, Denmark; 8 Department of Neurosurgery, Salford Royal NHS Foundation Trust, Salford M6 8HD, UK; 9 Department of Neurology, Salford Royal NHS Foundation Trust, Salford M6 8HD, UK

## Abstract

Epilepsy is a serious and common neurological disorder. Expression quantitative loci (eQTL) analysis is a vital aid for the identification and interpretation of disease-risk loci. Many eQTLs operate in a tissue- and condition-specific manner. We have performed the first genome-wide cis-eQTL analysis of human hippocampal tissue to include not only normal (*n* = 22) but also epileptic (*n* = 22) samples. We demonstrate that disease-associated variants from an epilepsy GWAS meta-analysis and a febrile seizures (FS) GWAS are significantly more enriched with epilepsy-eQTLs than with normal hippocampal eQTLs from two larger independent published studies. In contrast, GWAS meta-analyses of two other brain diseases associated with hippocampal pathology (Alzheimer’s disease and schizophrenia) are more enriched with normal hippocampal eQTLs than with epilepsy-eQTLs. These observations suggest that an eQTL analysis that includes disease-affected brain tissue is advantageous for detecting additional risk SNPs for the afflicting and closely related disorders, but not for distinct diseases affecting the same brain regions. We also show that epilepsy eQTLs are enriched within epilepsy-causing genes: an epilepsy cis-gene is significantly more likely to be a causal gene for a Mendelian epilepsy syndrome than to be a causal gene for another Mendelian disorder. Epilepsy cis-genes, compared to normal hippocampal cis-genes, are more enriched within epilepsy-causing genes. Hence, we utilize the epilepsy eQTL data for the functional interpretation of epilepsy disease-risk variants and, thereby, highlight novel potential causal genes for sporadic epilepsy. In conclusion, an epilepsy-eQTL analysis is superior to normal hippocampal tissue eQTL analyses for identifying the variants and genes underlying epilepsy.

## Background

Epilepsy is amongst the most common neurological disorders, affecting up to 1% of the population (1). Epilepsy is a complex genetic disease: hundreds, perhaps thousands, of genetic variants influence susceptibility to epilepsy (2). A number of genome-wide association studies (GWAS) and a GWAS meta-analysis have been conducted in order to identify genetic variants associated with common epilepsies, but have identified less than ten distinct loci (3). This lack of success is most readily explained by the limited statistical power of GWAS (4): many of the missing variants are likely to be hidden amongst the signals discarded for failing to meet the stringent threshold of statistical significance required to tackle the multiple testing burden.

Expression quantitative trait loci (eQTL) analysis is a tool for uncovering these hidden variants. eQTL studies are similar to traditional genetic-association studies, but instead of associating genetic variants with discrete traits such as disease status, eQTL studies correlate genetic variants with quantitative gene-expression levels. It is known that disease-associated variants are more likely to be eQTLs (5). By using this information, the discovery of disease-associated variants can be enhanced as association of a variant with gene expression lends credibility to its association with disease status. eQTL-based selection for validation has been shown to be a useful approach for identifying disease-risk loci from GWAS in a number of disorders, for example Crohn’s disease (6).

Another challenge in GWAS analysis is interpreting the biological relevance of susceptibility loci identified at genome-wide levels of significance: >90% of disease-associated variants from many GWAS lie in noncoding regions (7), making evaluation of their function difficult. However, the expression of a high percentage of genes is regulated by DNA variants (8–10), and >75% of significant GWAS variants map to regulatory regions in DNA (7,11). Hence, identifying a relationship between disease-associated genetic polymorphisms and gene expression levels offers potential insights into the functional impact of these variants.

Given that some eQTLs appear to be tissue-specific and many disease phenotypes manifest themselves only in certain tissues, the above approaches have been most successful when eQTL analyses have been performed in disease-relevant tissues. For example, eQTLs identified in immune (lymphoblastoid) cells showed greater enrichment in disease-risk variants for autoimmune diseases than in disease-risk variants for neurological disorders (5). In addition, the detection of some disease-relevant eQTLs requires the assessment of disease-affected tissue from subjects with the disease (12). Hence, eQTLs identified in coronary artery disease-affected tissues match more coronary artery disease-risk variants than eQTLs identified in the same tissue-types isolated from healthy individuals (13). Only two published eQTL studies have included samples from donors with brain diseases (alongside normal samples): one included tissue from donors with Alzheimer disease (14), while the other included tissue from donors with various psychiatric diseases (15). Neither of these studies assessed whether the inclusion of disease samples enhanced the identification of disease-risk variants.

We have performed the first genome-wide cis-eQTL analysis to include epileptic brain samples. Importantly, we have determined whether the inclusion of epileptic tissue enhances the identification of epilepsy disease-risk variants.

## Results

### Quality control and eQTL analysis overview

Three individuals were excluded from the eQTL analysis on the basis of genotyping QC filters, and one further individual was excluded from the eQTL analysis having failed the microarray QC criteria. We found no evidence of confounding effects from population stratification: the eigenvalues of none of the principal components reached statistical significance. Additionally, none of the probe expression profiles were significantly correlated with any of the top ten principal components, demonstrating that the gene expression profile was not significantly influenced by population stratification in this cohort (16). The final data-set, after quality control filtering, included 22 epilepsy cases and 22 normal controls, and 4 663 226 genotyped and imputed single nucleotide polymorphisms (SNPs). Patient characteristics are given in Supplementary Material, Table S1.

0.5% (15698/2900609) of all genotyped and imputed cis-SNPs were significant (FDR < 0.05) cis-eQTLs, and 3.95% of all analysed probes (748/18916) were significant (FDR < 0.05) cis-genes. MIAME-compliant array data has been deposited in a publically available database (ArrayExpress accession E-MTAB-3123). Full eQTL results are provided as a Supplementary Material.

### External validation of eQTL results

We determined if there was statistically significant replication between the results of our eQTL analysis (epilepsy-eQTL) and two independent published normal hippocampal tissue eQTL analyses: the Genotype-Tissue Expression Project Version 6 (17) analysis (GTEx) and the Braineac analysis (18). Replication with GTEx was ∼122 times greater for the significant than the non-significant SNP-gene pairs from the epilepsy-eQTL (Table 1). Replication with Braineac was ∼40 times greater for the significant than the non-significant SNP-gene pairs from the epilepsy-eQTL (Table 1). The degree of replication with both GTEx and Braineac was statistically significant (permutation-derived *P*-value <1 x 10^−4^). Overall, 19.3% (279 out of 1448) of the independent epilepsy-eQTL SNP-gene pairs were replicated in one of the validation datasets.

**Table 1:** Replication of epilepsy-eQTL gene-pairs

Validation eQTL study	Epilepsy-eQTL FDR	Total number of epilepsy-eQTL-gene pairs *(A)*	Overlap with validation eQTL-gene pairs *(B)*	% of *A* that overlaps with *B*	Relative ratio of significant over non-significant discovery eQTL-gene pairs that replicate with validation gene-pairs
GTEx	<0.05	764	121	15.84	122
	≥0.05	295 944	385	0.13	
Braineac	<0.05	719	3	0.40	40

### Enrichment of eQTL results within GWAS SNPs

**Figure 1:**
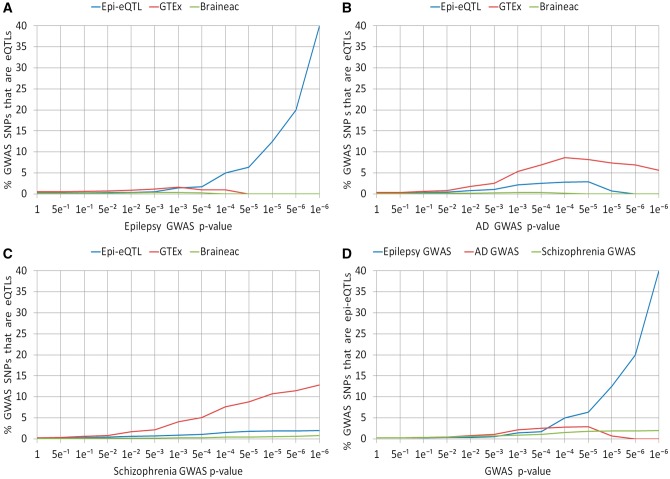
**(A–C)** Enrichment of significant SNPs from three eQTL studies (epilepsy-eQTL, GTEx and Braineac) within SNPs from three GWAS meta-analyses: epilepsy (A), Alzheimer’s disease (B) and schizophrenia (C). **(D)** Enrichment of significant SNPs from the epilepsy-eQTL within SNPs from the three GWAS meta-analyses. Epi-eQTL = epilepsy-eQTL; AD = Alzheimer’s disease; p-value notations as in the following example: 1e^**−**^^6^** **= 1 x 10^**−**^^6^.

Epilepsy GWAS meta-analysis SNPs (18) that are more strongly associated with the disease are more enriched with epilepsy-eQTLs (Fig. 1A and Supplementary Material, Table S2). At the point of maximal enrichment, epilepsy GWAS meta-analysis SNPs were ∼147 times more enriched with epilepsy-eQTLs than at baseline (GWAS *P*-value ≤1); this enrichment was statistically significant (permutation-based *P*-value <1x10^−4^). In comparison, epilepsy GWAS meta-analysis SNPs were ∼3 times more enriched with normal hippocampus eQTLs at the point of maximal enrichment than at baseline (GWAS *P*-value ≤1). The higher enrichment within epilepsy GWAS SNPs of epilepsy-eQTLs, relative to normal hippocampus eQTLs, was statistically significant (Supplementary Material, Table S3).

Febrile seizures (FS) GWAS (18) SNPs that are more strongly associated with the disease are more enriched with epilepsy-eQTLs (Supplementary Material, Fig. S1 and Supplementary Material, Table S4). At the point of maximal enrichment, FS GWAS SNPs were ∼125 times more enriched with epilepsy-eQTLs than at baseline (GWAS *P*-value <1); this enrichment was statistically significant (permutation-based *P*-value <1x10^−4^). In comparison, FS GWAS SNPs were ∼2 times more enriched with normal hippocampus eQTLs at the point of maximal enrichment than at baseline (GWAS *P*-value <1). The higher enrichment within FS GWAS SNPs of epilepsy-eQTLs, relative to normal hippocampus eQTLs, was statistically significant (Supplementary Material, Table S5).

On the other hand, the Alzheimer’s disease (AD)-(19) and schizophrenia-associated (20) GWAS meta-analyses SNPs that are more strongly associated with disease are more enriched with GTEx normal hippocampus eQTLs than with epilepsy-eQTLs (Fig. 1B–C and Supplementary Material, Table S2). Also, epilepsy-eQTLs were more enriched within GWAS meta-analyses SNPs associated with epilepsy than those associated with AD or schizophrenia (Fig. 1D and Supplementary Material, Table S2).

### Enrichment of GWAS SNPs within eQTL results

**Figure 2:**
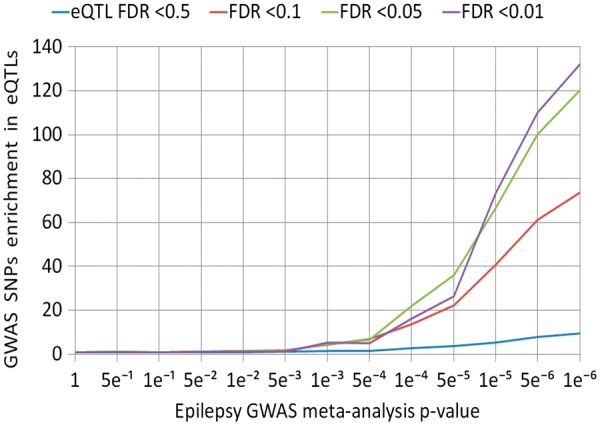
Relative enrichment of epilepsy GWAS meta-analysis SNPs within epilepsy-eQTL SNPs.

Significant epilepsy-eQTL SNPs showed increasing enrichment with epilepsy GWAS meta-analysis SNPs of progressively decreasing *P*-values (Fig. 2 and Supplementary Material, Table S6). In addition, more significantly associated epilepsy-eQTL SNPs were more enriched with epilepsy GWAS meta-analysis SNPs (Fig. 2 and Supplementary Material, Table S6). Similarly, significant epilepsy-eQTL SNPs showed increasing enrichment with FS GWAS SNPs of progressively decreasing *P*-values (Supplementary Material, Table S7), and more significantly associated epilepsy-eQTL SNPs were more enriched with FS GWAS SNPs (Supplementary Material, Table S7).

### Epilepsy eQTLs are enriched within Mendelian epilepsy genes

An epilepsy cis-gene is 2.6x more likely to be a Mendelian gene for epilepsy than a Mendelian gene for another disorder (Supplementary Material, Table S8). This enrichment of epilepsy eQTLs within causal genes for epilepsy is statistically significant (two-sided Fisher's exact test *P*-value < 0.05). In comparison, a Braineac cis-gene is 1.4x more likely to be a causal gene for epilepsy than to be a causal gene for another disorder, and a GTEx cis-gene is 0.5x less likely to be a causal gene for epilepsy than to be a causal gene for another disorder. The greater enrichment within Mendelian epilepsy genes of epilepsy cis-genes, compared to Braineac and GTEx cis-genes, is also statistically significant (permutation-based *P*-value < 0.05).

### Using epilepsy-eQTL results to aid interpretation of GWAS hits

One application of eQTL studies is to elucidate the genetic causes of disease through corroboration of GWAS disease-risk variants that achieve genome-wide level of significance, identification of disease-risk variants from amongst those failing to reach genome-wide level of significance, and functional interpretation of SNPs. Supplementary Material, Table S9 lists SNPs that achieved at least nominal level of significance (*P* < 0.05) in GWAS and were significant (FDR < 0.05) cis-eQTLs or were in strong LD (*r*^2^ > 0.8) with significant eQTLs. From this list, we highlight the most important examples below. 

rs35476054 achieved a genome-wide level of significance (*P*-value <3.99 x 10^−9^) in the epilepsy GWAS meta-analysis. rs35476054 is an independent signal not in LD with other genome-wide significant SNPs from the epilepsy GWAS meta-analysis. rs35476054 is not a known eQTL. rs35476054 is in tight LD (*r*^2^ = 0.92) with rs12992163, which is a located in and is an eQTL for *TTC21B* in our study, and leads to changes in multiple TF binding motifs according to ENCODE (21). This SNP also shows a suggestive association (*P*-value = 5 x 10^−7^) with febrile seizures in an independent GWAS (22). Another SNP from the latter GWAS, rs35049552, located just upstream of *TTC21B*, is significantly associated with febrile seizures (*P* < 1.5 x 10^−9^), and is in tight LD (*r*^2^ = 0.96) with rs9332423, which is also eQTL for *TTC21B* in the current study. rs9332423 leads to changes in TF binding motifs according to ENCODE, and is an eQTL for *TTC21B* in AD brain tissue (23). These disease SNPs are not in or in LD with SNPs from previous hippocampal eQTL studies (15,17,18). *TTC21B* knockdown mice exhibit major abnormalities in hippocampal structure (24). Also, *TTC21B* mutations cause ciliopathies (25), and ciliary dysfunction leads to neuronal excitability (26), spontaneous myoclonus (27) and susceptibility to pentylenetetrazol-induced seizures in mice (26,27). Ciliary dysfunction is seen in rodent models of epilepsy (28) and human ciliopathies are associated with seizures (29–32). Hence, our eQTL analysis reveals the potential functional impact of these significantly disease-associated SNPs, and shows that *TTC21B* is an important putative causal gene in both epilepsy and febrile seizures.

rs11086166 is nominally associated (*P* < 0.05) with disease in both the ILAE all epilepsy and focal epilepsy GWAS meta-analyses. This SNP lies between two very proximate genes, being 837 bp 5' of *KCNQ2* and 14 kb 3’ of *EEF1A2*, and is a significant (FDR < 0.05) cis-eQTL for both the genes in our analysis. There is independent external evidence that these genes are under shared regulatory control. Firstly, extensive microarray data collated within Genevestigator (33) shows that *KCNQ2* and *EEF1A2* expression levels are positively correlated across all human tissues (Pearson correlation coefficient *r* = 0.66), but more strongly correlated in the brain (*r* = 0.81) and even more so in the temporal lobe (*r* = 0.92). Hippocampal tissue constituted 61% (165/271) of the samples included in the temporal lobe analysis; an analysis restricted to hippocampal tissue was not enabled within Genevestigator. Secondly, there is evidence that both genes are co-regulated in the brain by another cis-eQTL, rs141448165, according to the Braineac eQTL analysis: rs141448165 is an eQTL for *KCNQ2* (nominal *P*-value = 1.9 x 10^−5^) and *EEF1A2* (nominal *P*-value = 3.0 x 10^−5^) in intralobular white matter. rs11086166 is in strong LD (*r*^2^ = 0.91) with rs884851 which is a cis-eQTL for *KCNQ2* in lymphoblastoid cells (34). Numerous distinct mutations and deletions affecting *KCNQ2* (35–51) or *EEF1A2* (52–57) or both (58,59) are known to cause Mendelian familial epilepsy syndromes or childhood epileptic encephalopathies. *KCNQ2* is a voltage-gated non-inactivating potassium ion channel that is downregulated in the epileptic human hippocampus (60,61). Drugs activating these channels reduce neuronal excitability and have antiepileptic efficacy (62). Retigabine, a positive allosteric modulator of *KCNQ2*, has recently been licenced for the treatment of epilepsy (63). *EEF1A2* is a tissue-specific translation elongation factor expressed only in neurons, cardiac and skeletal muscle (64). *EEF1A2-*mutant mice exhibit abnormal neurotransmitter uptake and release, demyelination and gliosis (64). 

rs11941935 is nominally associated (*P* < 0.05) with disease in the ILAE focal epilepsy GWAS meta-analysis. rs11941935 lies 3.9 kb 3’ of *LIAS* and is a significant (FDR < 0.05) cis-eQTL for this gene in our analysis. According to independent published studies, rs11941935 is a significant cis-eQTL for LIAS in blood (34,65,66), skin (34) and adipocytes (34). Different mutations in LIAS cause severe syndromes characterized by the development of seizures, epilepsy and epileptic encephalopathy (67,68). *LIAS*-mutant mice display prominent abnormalities in brain morphology (69).

The above observations suggests that genetic variation in *KCNQ2, EEF1A2* and *LIAS*, known to cause familial epilepsy syndromes or epileptic encephalopathies, might also play a role in the development of sporadic focal epilepsy.

Analysis of the epilepsy GWAS and eQTL results using Sherlock (70), a tool which detects disease genes by integrating GWAS and eQTL data, corroborated the association between *TTC21B* and epilepsy (Log Bayes Factor = 5.45, *P*-value = 1.7 x 10^−5^, FDR = 0.01). Other genes showed more modest associations in the Sherlock analysis (*P*-values ≥0.001). These findings are in keeping with previously reported results from the creators of Sherlock: only one gene met their predefined criteria of *P* < 1 x 10^−4^ (FDR < 0.09) for association with Crohn's disease, when considering cis-SNPs only (70). Complete results from our Sherlock analysis are detailed in Supplementary Material, Table S10.

## Discussion

The contributions of this study are 3-fold. Firstly, this study objectively demonstrates that epilepsy disease-risk SNPs are significantly more enriched in an eQTL analysis of disease-affected brain tissue from people with epilepsy than in eQTL analyses of the same tissue types from unaffected donors. Secondly, we use the results of our disease-specific eQTL analysis to provide novel insights into the genetic causes of epilepsy. Thirdly, the full epilepsy-eQTL results are released as a novel resource to aid the interpretation of other findings from current and future GWAS analyses of epilepsy.

It is known that eQTLs identified in disease-relevant tissue types from normal donors are more enriched in disease-risk loci than eQTLs identified in non-relevant tissue types (5). In addition, it has been suggested that including in the eQTL analysis disease-tissue from donors with the disease enhances the detection of disease-risk variants. A recent study found that eQTLs identified in coronary artery disease-affected tissues match more coronary artery disease-risk variants than eQTLs identified in the same tissue-types isolated from healthy individuals, although they did not determine if the difference is statistically significant (13). We are not aware of a similar published analysis with brain tissue eQTLs. As with our analysis, Webster *et al.* (14) included disease-relevant tissue from donors with and without the disease. They showed that inclusion of tissue from individuals with the disease led to the detection of some eQTLs not otherwise identified. The authors did not determine, however, if this strategy leads to the identification of more disease-associated variants. Intuitively, the inclusion of both normal and pathological tissue should enhance the ability to detect disease-related eQTLs as the datasets analysed will capture the transcriptomic and genotypic differences between patients and controls. Hence, we performed a genome-wide cis-eQTL analysis that included epileptic human hippocampal tissue. We found that this epilepsy-eQTL analysis is superior to normal hippocampal tissue eQTL analyses for the detection of epilepsy disease-risk loci: epilepsy-eQTLs were significantly more enriched with epilepsy disease-risk variants than eQTLs from larger normal hippocampal analyses (Fig. 1A). These findings are unlikely to be spurious as epilepsy-eQTLs were significantly more enriched than normal hippocampal tissue eQTLs in a completely independent GWAS of FS. To further demonstrate that eQTLs identified in disease-effected tissue are of most relevance to the afflicting disease, we also showed that disease-risk loci for two brain diseases with proven hippocampal involvement, AD (71) and schizophrenia (72), were less enriched with hippocampal epilepsy-eQTLs than with normal hippocampal eQTLs (Fig. 1B–D). These observations suggest that an eQTL analysis of disease-affected brain tissue is advantageous for detecting additional risk SNPs for the afflicting and closely related diseases, but not for distinct disorders affecting the same brain regions. This highlights the need to perform disease-specific eQTL analyses in other brain diseases.

We studied the relationship of epilepsy eQTLs to known human disease genes that cause familial Mendelian disorders. We found that epilepsy eQTLs are enriched within Mendelian epilepsy genes: an epilepsy cis-gene is significantly more likely to be a causal gene for a Mendelian epilepsy syndrome than to be a causal gene for another Mendelian disorder. The enrichment within causal epilepsy genes of epilepsy cis-genes, compared to that of Braineac and GTEx cis-genes, is greater than expected by chance alone. It has been shown recently that genes known to cause Mendelian familial forms of a disease can contribute to sporadic forms of the disease (73). Hence, these observations lend support to the disease-specific pathogenic nature of epilepsy eQTLs. There is an increasing interest in identification of functionally relevant genes through integration of GWAS and eQTL summary results, and specialized analytical approaches are being developed for this purpose (74). Such approaches may benefit from the use of disease-specific eQTL results; this should be investigated in future studies.

One of the applications of eQTL studies is to reveal disease-causing genes by identifying cis-SNPs that corroborate GWAS disease-risk variants and then link them to cis-genes of potential causal relevance. In epilepsy, as with other complex genetic diseases, for example, schizophrenia, thousands of genetic variants potentially influence disease susceptibility (2). A comprehensive recent epilepsy GWAS meta-analysis, which included 8696 cases, reported two independent loci at genome-wide level of significance (4). Many of the missing variants are likely to be hidden amongst the signals discarded for failing to meet the stringent threshold of statistical significance required to tackle the multiple testing burden. eQTL studies, such as this one, that include epileptic tissue are vital to the identification of novel epilepsy disease-risk variants from amongst SNPs failing to reach genome-wide level of significance. Supplementary Material, Table S9 lists SNPs that achieved at least nominal level of significance (*P* < 0.05) in GWAS and were significant (FDR < 0.05) epilepsy-eQTLs, along with their respective cis-genes. As larger multinational epilepsy GWAS collaborations that are currently in progress (www.epipgx.eu) start to generate results, this list of potentially causal SNPs will increase, as will the utility of our epilepsy-eQTL analysis.

Examining individual GWAS variant-eQTL associations, an important finding of our study is that a top epilepsy SNP and a top FS SNP, both associated with their respective phenotype at genome-wide level of significance, are cis-eQTLs for *TTC21B*. Hence, *TTC21B* is potentially an important causal gene underlying both epilepsy and FS. This assertion is supported by the following observations. These cis-SNPs lead to changes in multiple TF binding motifs (21), there is corroboration of at least one of the cis-eQTLs from an independent analysis (22), and *TTC21B* mutations cause major hippocampal morphological abnormalities in rodents (24) and ciliary dysfunction (25), which promotes seizures in animal models and man (26,27–32). Given the paucity of statistically significant gene associations with sporadic epilepsy, *TTC21B* is an especially valuable addition.

Another notable finding is that some nominally disease-associated SNPs are significant eQTLs for genes implicated in monogenic Mendelian epilepsy syndromes: *KCNQ2*, *EEF1A2* and *LIAS*. A number of observations make these compelling potential causal genes in sporadic epilepsy. The eQTL associations are corroborated by data from independent published studies (34,65,66). The effect of their dysfunction in animal models is congruent with enhanced seizure susceptibility (64,69). Many different mutations and deletions affecting these genes produce a number of syndromes characterized by the development of seizures, epilepsy and epileptic encephalopathy (35–59,67,68). It has been shown recently that genes known to cause Mendelian familial forms of a disease can contribute to sporadic forms of the disease (73). Indeed, one of the associations reported by the all epilepsy GWAS meta-analysis was with *SCN1A* (4), which is implicated in some monogenic epilepsies and is targeted by a number of conventional antiepileptic drugs (75). These points indicate that *KCNQ2*, *EEF1A2* and *LIAS* are particularly promising leads in the search for causal genes and therapeutic targets in sporadic epilepsy. Indeed, *KCNQ2* is targeted by a drug recently licenced for the treatment of epilepsy, retigabine, which is efficacious is sporadic epilepsy (63).

It might be deemed intuitive and expected that an eQTL analysis that includes disease-affected brain tissue will be more disease-relevant. However, demonstrating this objectively is of value as it evidences and stresses the need to include disease-affected brain tissue in future eQTL studies and the advantages of such a strategy. We acknowledge that we have analysed a relatively small number of samples. We would advocate future epilepsy-eQTL analyses with larger numbers of samples in order to confirm our findings and to discover new eQTLs. We are aware of a recent study that included a limited trans-eQTL analysis of epileptic brain tissue in order to identify SNPs regulating specific gene co-expression modules that they had deemed to be important (76); that study does not include genome-wide cis-eQTL SNPs and targets.

## Conclusions

In conclusion, we have presented the first genome-wide cis-eQTL analysis to include epileptic human hippocampal tissue. We have shown that this epilepsy-eQTL analysis is superior to normal hippocampal tissue eQTL analyses for the detection and interpretation of epilepsy disease-risk variants and genes, and provides novel insights into the genetic causes of epilepsy. Our results highlight the need to perform disease-specific eQTL studies in other brain diseases and to perform larger epilepsy-eQTL studies.

## Materials and Methods

### Collection of human hippocampal samples, RNA isolation, microarray processing, and microarray data normalization, adjustment and filtering

A brief summary of these steps is presented here; a detailed description can be found in our previous publication (60). The study was approved by the Northwest 2 Research Ethics Committee. Informed consent was obtained from tissue donors. Epileptic hippocampal samples were obtained from patients undergoing therapeutic resection for refractory mesial temporal lobe epilepsy. Frozen post-mortem histologically-normal hippocampal samples from donors with no known brain diseases were obtained from tissue banks. RNA isolated from hippocampal samples was analysed using Agilent SurePrint G3 Custom 8x60K Microarrays designed to contain probes for all known genes. Batch effect was corrected using the ComBat (77) algorithm. To adjust for known and unknown confounders, Independent Surrogate Variable Analysis (78) was applied; RIN, age and sex were explicitly included in the ISVA adjustment. Where multiple probes mapped to the same gene, the probe with the highest variance was retained, as the most variant probe is likely to be most informative.

Polymorphisms present in the probe-target sequences have been shown to alter probe hybridization affinities, leading to reduced signal intensity measurements and resulting in false-positive results (79). We used the ‘PIP Finder’ tool (79) to determine which probes in our microarray had polymorphisms with a minor allele frequency >1% in Europeans located within the probe sequences; all such probes were excluded from the eQTL analysis in keeping with current practice.

### DNA isolation, genotyping, quality control and imputation

DNA was extracted from brain samples using the DNeasy Blood & Tissue Kit (Qiagen, Crawley, United Kingdom) according to the manufacturer’s recommended protocol. All samples were genotyped on the Illumina Infinium HumanOmniExpressExome BeadChip in the ARK Genomics facility at the Roslin Institute, UK. Per-SNP and per-individual quality control (QC) procedures were applied, as detailed in the Supplementary Material.

Genotype imputation was performed using Impute2 (80), with 1000 Genomes Project haplotypes (March 2012) as the reference panel. The QCTOOL software was used to exclude SNPs with an ‘info’ score of <0.9. GTOOL software was then used to transform the imputed data to PED format, with a posterior probability of 0.9 used as a threshold to call genotypes. Our standard per-SNP QC filters, as detailed in the Supplementary Material, were then applied.

### eQTL analysis

eQTL analysis was performed using the linear model in the program Matrix eQTL (81). We defined cis-SNPs as those within 20 kb of the target gene. There is no consensus (82), amongst researchers, on the distance cut-off relative to the regulated gene used to define cis-SNPs, with distances ranging from 20 kb (83–86) to 1 Mb (87), or even longer (88,89), being found in published literature. We opted for the conservative end of this range as cis-eQTLs rarely reside more than 20 kb away from the gene (90), and cis-eQTL effect sizes (91,92) and replication rates (18) decay (and, inversely, false positives increase) with increasing distance from the target gene. Threshold of statistical significance was set at Benjamini–Hochberg False Discovery Rate (FDR) < 0.05. To investigate potential confounding effects from population stratification, the principal components (PC) of variance of the genotyping dataset were calculated using the package SNPRelate (93), and then the Tracy-Widom (TW) statistic (94) and significance for the eigenvalue of each PC was calculated using the package EigenCorr (95). Furthermore, the top ten PCs were separately correlated with the probe expression profiles in R.

### Datasets for validation and enrichment analyses

For validation, hippocampal eQTLs were extracted from the published results of the Genotype-Tissue Expression Project Version 6 (GTEx) (17) and the Braineac study (18); these are the largest eQTL analyses of normal human hippocampal tissue published to date, including 81 and 134 samples, respectively. For enrichment analyses, complete results from the largest GWAS meta-analyses of epilepsy (4), Alzheimer’s disease (AD) (19) and schizophrenia (20) were downloaded. Complete results from a recent GWAS of febrile seizures (FS) (22) were also used for enrichment analysis; these results have not been publicly released, hence, scripts were provided to the authors and run on the FS GWAS results by them.

### External validation of eQTL results

We determined if there is statistically significant replication between the results of our eQTL analysis (epilepsy-eQTL) and the GTEx normal human hippocampus eQTL analysis. We determined the fraction of statistically significant (FDR <0.05) SNP-gene pairs from the epilepsy-eQTL that were replicated in GTEx. For comparison, we determined the fraction of non-significant (FDR ≥0.05) SNP-gene pairs from the epilepsy-eQTL that were replicated in GTEx. For these comparative analyses, we included only the SNPs analysed in both eQTL studies, and LD-pruned the SNPs. To enable comparison between the two studies, all gene identifiers were converted to Entrez Gene IDs. To determine the statistical significance of the replication, a permutation-based approach was adopted, which is detailed in Supplementary Material. Briefly, 10 000 sets of randomly-chosen epilepsy-eQTL SNP-gene pairs were created and their overlap with significant SNP-gene pairs from GTEx was determined.

Using the aforementioned methods, we also determined if there is statistically significant replication between the results of the epilepsy-eQTL and the Braineac eQTL analysis. It should be noted that the authors of Braineac have provided only LD-pruned eQTL results; these were used to determine the statistical significance of replication with the epilepsy-eQTL.

The total number of independent epilepsy-eQTLs that were replicated in either the GTEx or Braineac analyses was also calculated. We created an LD-pruned list of epilepsy-eQTL SNP-gene pairs. Epilepsy-eQTL SNP-gene pairs also found in one of the validation eQTLs were deemed replicated. Also considered replicated were epilepsy-eQTL SNP-gene pairs where the SNP was in high LD (*r*^2^ > 0.8) with a different SNP that was an eQTL for the same gene in one of the validation datasets.

### Enrichment of eQTL results within GWAS SNPs

Enrichment of eQTL results within GWAS SNPs was calculated using previously published methodology (96,97), which is detailed in the Supplementary Material. Briefly, a list of SNPs included both in the ILAE GWAS meta-analysis and the epilepsy-eQTL was created. This list was LD-pruned. For the GWAS SNPs that remained after LD-pruning, the fraction that were significant eQTLs was calculated for a range of GWAS *P*-values. At the point of maximal enrichment, the statistical significance of the overlap between disease SNPs and eQTLs was calculated using a permutation-based approach. Another permutation-based analysis was completed in order to determine if the higher enrichment of the epilepsy-eQTL SNPs, compared to GTEx SNPs, within the epilepsy GWAS was statistically significant (please see Supplementary Material).

The above processes were repeated for the epilepsy-eQTL and Braineac analyses. The above analyses were also repeated for the FS GWAS.

### Enrichment of GWAS SNPs within eQTL results

We determined if significant epilepsy-eQTLs were enriched with significant epilepsy GWAS meta-analysis SNPs. A list of SNPs included both in the ILAE GWAS meta-analysis and the epilepsy-eQTL analysis was created and LD-pruned. From this list, we calculated the fraction (*f1*) of SNPs with GWAS *P*-value <1 x 10^−6^. We created a subset of SNPs with epilepsy-eQTL FDR < 0.05, and calculated the fraction (*f2*) of SNPs with GWAS *P*-value <1 x 10^−6^ in this subset. The relative enrichment of significant epilepsy GWAS meta-analysis SNPs within significant epilepsy-eQTLs was defined as *f2*/*f1*. These calculations were repeated for a range of epilepsy GWAS meta-analysis *P*-values and epilepsy-eQTL FDRs. In the rare situation that a SNP appeared more than once in the epilepsy-eQTL results, the lowest FDR for the SNP was used.

### Enrichment of eQTLs within human disease genes

We studied the relationship of epilepsy eQTLs to known human disease genes that cause familial Mendelian disorders. Genes known to cause Mendelian human diseases were extracted from the Online Mendelian Inheritance in Man (OMIM) Morbid Map (omim.org/downloads; date last accessed September 01, 2016) and mapped to Entrez gene identifiers. Within this list, genes that cause Mendelian epilepsy syndromes—disorders in which epilepsy or seizures are a cardinal feature—were identified. The enrichment of epilepsy eQTLs within causal genes for epilepsy, compared to causal genes for other diseases, was determined. Specifically, the proportion of Mendelian epilepsy genes that are epilepsy cis-genes, and the proportion of other Mendelian genes that are epilepsy cis-genes was calculated. The two proportions were compared using a two-sided Fisher’s exact test, with the level of statistical significance set at *P*-value < 0.05.

Using the above procedure, the enrichment of Braineac eQTLs within Mendelian epilepsy genes and the enrichment of GTEx eQTLs within Mendelian epilepsy genes was calculated. Within Mendelian epilepsy genes, epilepsy cis-genes were enriched more than Braineac and GTEx cis-genes; the statistical significance of the relative enrichment was determined using permutation-based analysis (please see Supplementary Material).

### Functional interpretation of GWAS results

In order to demonstrate the utility of the epilepsy-eQTL dataset for the functional interpretation of specific findings from relevant GWAS analyses, we identified examples of variant(s) failing to reach genome-wide level of significance in the epilepsy GWAS meta-analysis but revealed to be putatively functionally relevant using the epilepsy-eQTL results, and variant(s) reaching genome-wide level of significance in the epilepsy GWAS meta-analysis whose functional relevance and effect is revealed only by utilizing epilepsy-eQTLs. ENCODE data was interrogate using HaploReg (19) in order to determine if eQTLs produce changes in transcription factor (TF) binding motifs. Genevestigator (33) co-expression tool was used to determine the similarity between the transcriptomic profiles of *KCNQ2* and *EEF1A2* (see Results).

Epilepsy GWAS meta-analysis and epilepsy eQTL results were submitted to Sherlock (http://sherlock.ucsf.edu/; date last accessed December 11, 2016) (70), an analytical tool for detecting disease genes by integrating eQTL and GWAS data.

## Supplementary Material

Supplementary Material is available at *HMG* online.

## Supplementary Material

Supplementary Data
