# Supplementary material for: Genetic regulation of gene expression in the epileptic human hippocampus
Source: Hum Mol Genet. 2017 Mar 3;26(9):1759–69. doi: 10.1093/hmg/ddx061 (PMC5411756; doi:10.1093/hmg/ddx061)
Supplement: Supplementary Data [file ddx061_Supp.zip › HMG_eQTL_SupplementalMaterial_revised_2.docx]

Table of Contents

[1. Supplemental Methods 2](#_Toc460849122)

[1.1 Genotyping data quality control criteria 2](#_Toc460849123)

[1.2 Permutation-analysis to determine statistical significance of replication between epilepsy-eQTLs and validation eQTLs 2](#_Toc460849124)

[1.3 Enrichment of eQTL results within GWAS SNPs 2](#_Toc460849125)

[1.4 Enrichment of epilepsy eQTLs within causal epilepsy genes 3](#_Toc460849126)

[2. Supplemental Results: Supplemental Tables 5](#_Toc460849127)

[3. Supplemental Results: Supplemental Figures 21](#_Toc460849128)

[4. Full eQTL results 21](#_Toc460849129)

[5. References 22](#_Toc460849130)

# 1. Supplemental Methods

## 1.1 Genotyping data quality control criteria

DNA was extracted from brain samples using the DNeasy Blood & Tissue Kit (Qiagen, Crawley, United Kingdom) according to the manufacturer’s recommended protocol. All samples were genotyped on the Illumina Infinium HumanOmniExpressExome BeadChip in the ARK Genomics facility at the Roslin Institute, UK. Our quality control (QC) procedures were as follows. Individuals were excluded on the basis of discordance between reported and observed sex, SNP call rate <95%, outlying autosomal heterozygosity rates (>3 standard deviations from the mean), inadvertent subject duplication or cryptic relatedness based on pairwise identity-by-descent estimations (the individual with greater missing data was removed from each pair with π-hat >0.1875), being outliers from the European cluster in a plot of the first two principal components of genotypes for samples from the study and HapMap Phase III. SNPs were excluded on the basis of genotype missing rate ≥5% if minor allele frequency (MAF) was ≥0.05 and missing rate ≥1% if MAF was <0.05, observed minor allele frequency <0.01, significant deviation (P <10^−4^) from Hardy-Weinberg equilibrium, significant difference (P <10^−4^) in the genotype missing rate in normal compared to epileptic samples, and all non-autosomal SNPs.

## 1.2 Permutation-analysis to determine statistical significance of replication between epilepsy-eQTLs and validation eQTLs

We determined if there is statistically significant replication between the results of our eQTL analysis (epilepsy-eQTL) and the GTEx normal human hippocampus eQTL analysis. For each probe included in the epilepsy-eQTL analysis, the most strongly associated SNP was chosen, forming a unique SNP-gene pair. We determined the fraction of statistically significant (FDR <0.05) SNP-gene pairs from the epilepsy-eQTL that were replicated in GTEx. For comparison, we determined the fraction of non-significant (FDR ≥0.05) SNP-gene pairs from the epilepsy-eQTL that were replicated in GTEx. For these comparative analyses, we included only the SNPs analysed in both eQTL studies. To enable comparison between the two studies, all gene identifiers were converted to Entrez Gene IDs. To determine the statistical significance of the replication, a permutation-based approach was adopted:

1. a list of all SNP-gene pairs *x* was created from the epilepsy-eQTL: *x* is the complete list of all significant and non-significant SNP-gene pairs, when considering only the list of SNPs included in both eQTL analyses;
2. from *x*, *y* number of SNP-gene pairs were randomly selected; *y* is the number of significant (FDR <0.05) SNP-gene pairs within *x*;
3. the size of the overlap between *y* and the significant SNP-gene pairs from GTEx was determined;
4. the above steps were repeated 10,000 times.

## 1.3 Enrichment of eQTL results within GWAS SNPs

Enrichment of eQTL results within GWAS SNPs was calculated using previously published methodology ([1](#_ENREF_1), [2](#_ENREF_2)). A list of SNPs included both in the ILAE GWAS meta-analysis and the epilepsy-eQTL was created. This list was LD-pruned using plink 1.9 ([3](#_ENREF_3)) (--indep-pairwise 1000kb 100 0.8), with the 1000 Genomes Project European Phase 3 Release ([4](#_ENREF_4)) as the reference dataset. For the GWAS SNPs that remained after LD-pruning, the fraction that were significant eQTLs was calculated for a range of GWAS p-values. When counting the number of GWAS SNPs that are also eQTLs, all GWAS SNPs that are significant eQTLs or in high LD (r^2^ >0.8) with significant eQTLs were included. At the point of maximal enrichment, the statistical significance of the overlap between disease SNPs and eQTLs was calculated using the following permutation-based approach:

1. 10,000 random sets of LD-pruned and MAF-matched GWAS SNPs were created, where the size of each random set is equal to the number of disease SNPs at the GWAS p-value;
2. the size of overlap with eQTLs was calculated for each random set;
3. the number of random sets with an overlap ≥ the size of the overlap between disease SNPs and eQTLs was determined.

The above processes were repeated for the AD and schizophrenia GWAS meta-analyses.

In order to determine if the higher enrichment of the epilepsy-eQTL SNPs, compared to GTEx SNPs, within the epilepsy GWAS was statistically significant, the following permutation-based approach was adopted:

1. an LD-pruned list of SNPs included in the GWAS, the epilepsy-eQTL and the GTEx analyses was created;
2. for disease SNPs at various GWAS p-values, the *overlap differential* was calculated, where the *overlap differential* = (size of overlap with epilepsy-eQTLs) – (size of overlap with Braineac eQTLs);
3. 10,000 random sets of LD-pruned GWAS SNPs were created, where the size of each random set is equal to the number of disease SNPs at a given GWAS p-value;
4. for each random set, the overlap differential was calculated;
5. the number of random sets with an overlap differential ≥ the actual overlap differential for that GWAS p-value was determined;
6. as this permutation analysis is solely comparative, with the same random SNP sets being examined for overlap with the two eQTL studies, the random SNPs were not required to be MAF-matched with the disease SNPs.

The above processes were repeated for the epilepsy-eQTL and Braineac analyses. The above analyses were repeated for the FS GWAS.

## 1.4 Enrichment of epilepsy eQTLs within causal epilepsy genes

Within Mendelian epilepsy genes, epilepsy cis-genes were enriched more than Braineac and GTEx cis-genes; the statistical significance of the relative enrichment was determined using a permutation-based analysis, described below. Only genes with official Entrez gene identifiers were included in the analysis.

The number of cis-genes identified by the epilepsy-eQTL analysis (577) was less than that identified by the Braineac (1197) and GTEx (814) eQTL analyses. Hence, the overlap with Mendelian disease genes was less for epilepsy cis-genes (112) than for Braineac (230) and GTEx (183) cis-genes. Relative to their overlap with all Mendelian disease genes, the overlap with Mendelian epilepsy genes was greater for epilepsy cis-genes (6) than for Braineac (7) and GTEx (2) cis-genes. Using a permutation-based approach, we determined the probability that the same or greater relative enrichment of epilepsy cis-genes above the other cis-genes could occur by chance:

There were 6231 unique Mendelian disease genes, of which 134 were unique Mendelian epilepsy genes (Table S8). Mendelian epilepsy genes overlapped with 6 epilepsy cis-genes, 7 Braineac cis-genes and 2 GTEx cis-gene. All 6231 Mendelian disease genes were labelled as:

*A*: epilepsy cis-genes (112 genes),

*B*: Braineac cis-genes (230 genes),

*C*: GTEx cis-genes (183 genes).

134 genes were randomly selected from the full list of 6231 Mendelian disease genes and the number of *A*s, *B*s and *C*s was counted. This process was repeated 10,000 times. The number of random selections with ≥6 *A*s and ≤7 *B*s and ≤2 *C*s was used to determine the probability that the same or greater relative enrichment of epilepsy cis-genes above the other cis-genes could occur by chance alone.

# 2. Supplemental Results: Supplemental Tables

Table S1 Patient characteristics. RIN=RNA Integrity Number. The sample labels used in our previous publication ([5](#_ENREF_5)) have been maintained.

| **Sample** | **Phenotype** | **Age** | **Sex** | **RIN** |  | **Sample** | **Phenotype** | **Age** | **Sex** | **RIN** |
| --- | --- | --- | --- | --- | --- | --- | --- | --- | --- | --- |
| **D1** | epilepsy | 41 | F | 7.4 |  | **N1** | normal | 81 | M | 6.5 |
| **D2** | epilepsy | 23 | F | 6.8 |  | **N2** | normal | 78 | F | 6.2 |
| **D3** | epilepsy | 51 | M | 6 |  | **N3** | normal | 84 | F | 6.6 |
| **D4** | epilepsy | 49 | F | 6.9 |  | **N4** | normal | 91 | F | 6.7 |
| **D5** | epilepsy | 50 | F | 7.8 |  | **N5** | normal | 88 | M | 6.3 |
| **D6** | epilepsy | 45 | F | 6.6 |  | **N6** | normal | 38 | M | 6.1 |
| **D7** | epilepsy | 12 | M | 7 |  | **N7** | normal | 50 | M | 6.2 |
| **D8** | epilepsy | 29 | F | 7.8 |  | **N8** | normal | 45 | M | 6.3 |
| **D9** | epilepsy | 33 | M | 6.8 |  | **N9** | normal | 39 | M | 6.1 |
| **D11** | epilepsy | 34 | M | 7 |  | **N10** | normal | 40 | M | 6 |
| **D12** | epilepsy | 33 | M | 8.8 |  | **N11** | normal | 61 | M | 6.2 |
| **D14** | epilepsy | 22 | F | 7.3 |  | **N12** | normal | 63 | F | 6.2 |
| **D15** | epilepsy | 48 | M | 7.6 |  | **N13** | normal | 66 | M | 6.2 |
| **D16** | epilepsy | 39 | F | 7.4 |  | **N14** | normal | 22 | F | 6.3 |
| **D17** | epilepsy | 29 | F | 7.1 |  | **N15** | normal | 27 | M | 6.3 |
| **D18** | epilepsy | 44 | M | 7.9 |  | **N16** | normal | 45 | M | 6.9 |
| **D19** | epilepsy | 40 | F | 6.6 |  | **N18** | normal | 50 | M | 6.5 |
| **D20** | epilepsy | 48 | M | 8.5 |  | **N19** | normal | 43 | M | 6.6 |
| **D21** | epilepsy | 23 | M | 8.4 |  | **N20** | normal | 46 | M | 6.8 |
| **D22** | epilepsy | 63 | M | 7.9 |  | **N21** | normal | 51 | M | 6.2 |
| **D23** | epilepsy | 31 | M | 8.2 |  | **N22** | normal | 48 | M | 6 |
| **D24** | epilepsy | 27 | F | 8.2 |  | **N23** | normal | 43 | M | 6.6 |

MIAME-compliant data has been deposited in a publically available database (ArrayExpress accession E-MTAB-3123). Please note that the following samples included in E-MTAB-3123 were not used in the eQTL analysis: 12, 28, 36.

**Table S2** Percentage of SNPs from three GWAS meta-analyses (epilepsy, Alzheimer’s disease and schizophrenia) that are significant SNPs in three eQTL studies (epilepsy-eQTL, GTEx and Braineac). Epi-eQTL = epilepsy-eQTL; AD = Alzheimer’s disease.

| GWAS p-value | Epilepsy GWAS | | | AD GWAS | | | Schizophrenia GWAS | | |
| --- | --- | --- | --- | --- | --- | --- | --- | --- | --- |
|  | Epi-eQTL | GTEx | Brain--eac | Epi-eQTL | GTEx | Brain-eac | Epi-eQTL | GTEx | Brain--eac |
| 1 | 0.3 | 0.5 | 0.1 | 0.2 | 0.3 | 0.1 | 0.2 | 0.3 | 0.1 |
| 5x10^−1^ | 0.3 | 0.5 | 0.1 | 0.2 | 0.4 | 0.1 | 0.3 | 0.3 | 0.1 |
| 1x10^−1^ | 0.3 | 0.6 | 0.1 | 0.4 | 0.6 | 0.1 | 0.3 | 0.6 | 0.1 |
| 5x10^−2^ | 0.3 | 0.7 | 0.1 | 0.5 | 0.8 | 0.1 | 0.4 | 0.8 | 0.1 |
| 1x10^−2^ | 0.4 | 0.9 | 0.2 | 0.8 | 1.8 | 0.2 | 0.6 | 1.7 | 0.2 |
| 5x10^−3^ | 0.5 | 1.1 | 0.3 | 1.1 | 2.6 | 0.2 | 0.7 | 2.2 | 0.2 |
| 1x10^−3^ | 1.4 | 1.6 | 0.3 | 2.1 | 5.3 | 0.3 | 0.8 | 4.0 | 0.2 |
| 5x10^−4^ | 1.7 | 1.0 | 0.3 | 2.6 | 6.9 | 0.3 | 1.1 | 5.1 | 0.3 |
| 1x10^−4^ | 5.0 | 0.9 | 0.0 | 2.8 | 8.6 | 0.2 | 1.5 | 7.6 | 0.4 |
| 5x10^−5^ | 6.4 | 0.0 | 0.0 | 2.9 | 8.2 | 0.0 | 1.8 | 8.8 | 0.4 |
| 1x10^−5^ | 12.5 | 0.0 | 0.0 | 0.7 | 7.4 | 0.0 | 1.9 | 10.8 | 0.5 |
| 5x10^−6^ | 20.0 | 0.0 | 0.0 | 0.0 | 6.9 | 0.0 | 1.9 | 11.5 | 0.6 |
| 1x10^−6^ | 40.0 | 0.0 | 0.0 | 0.0 | 5.7 | 0.0 | 2.0 | 12.9 | 0.8 |

Table S3 Higher enrichment of epilepsy-eQTLs, compared to other eQTLs, within epilepsy GWAS meta-analysis SNPs: calculation of statistical significance by permutation.

| GWAS meta-analysis p-value | Permutation-based p-value (x10^−4^) | |
| --- | --- | --- |
|  | Braineac | GTEx |
| <1x10^-3^ | 44 | 538 |
| <5x10^-4^ | 19 | 16 |
| <1x10^-4^ | 4 | 4 |
| <5x10^-5^ | 1 | 1 |
| <1x10^-5^ | <1 | 1 |
| <5x10^-6^ | <1 | <1 |
| <1x10^-6^ | <1 | <1 |

Table S4 Percentage of SNPs from FS GWAS that are significant SNPs in three eQTL studies (epilepsy-eQTL, GTEx and Braineac). Epi-eQTL = epilepsy-eQTL

| GWAS p-value | Epi-eQTL | GTEx | Braineac |
| --- | --- | --- | --- |
| 1 | 0.2 | 0.3 | 0.1 |
| 5x10^−2^ | 0.3 | 0.4 | 0.1 |
| 1x10^−1^ | 0.3 | 0.5 | 0.1 |
| 5x10^−2^ | 0.3 | 0.5 | 0.1 |
| 1x10^−2^ | 0.4 | 0.6 | 0.1 |
| 5x10^−3^ | 0.4 | 0.5 | 0.1 |
| 1x10^−3^ | 1.0 | 0.2 | 0.1 |
| 5x10^−4^ | 1.6 | 0.0 | 0.2 |
| 1x10^−4^ | 4.4 | 0.0 | 0.0 |
| 5x10^−5^ | 7.2 | 0.0 | 0.0 |
| 1x10^−5^ | 14.3 | 0.0 | 0.0 |
| 5x10^−6^ | 18.2 | 0.0 | 0.0 |
| 1x10^−6^ | 25.0 | 0.0 | 0.0 |

Table S5 Higher enrichment of epilepsy-eQTLs, compared to other eQTLs, within FS GWAS SNPs: calculation of statistical significance by permutation.

| GWAS meta-analysis p-value | Permutation-based p-value (x10^−4^) | |
| --- | --- | --- |
|  | Braineac | GTEx |
| <1x10^-3^ | 47 | 15 |
| <5x10^-4^ | 1 | <1 |
| <1x10^-4^ | <1 | <1 |
| <5x10^-5^ | <1 | <1 |
| <1x10^-5^ | <1 | <1 |
| <5x10^-6^ | <1 | <1 |
| <1x10^-6^ | <1 | <1 |

Table S6 Fold difference of the fraction of epilepsy-eQTL SNPs that are disease-associated relative to the fraction of all SNPs that are disease-associated. FDR = false discovery rate

| Epilepsy GWAS  meta-analysis p-value | Epilepsy-eQTL FDR | | | |
| --- | --- | --- | --- | --- |
|  | <0.5 | <0.1 | <0.05 | <0.01 |
| 1 | 1 | 1 | 1 | 1 |
| 5x10^−1^ | 1 | 1 | 1 | 1 |
| 1x10^−1^ | 1 | 1 | 1 | 1 |
| 5x10^−2^ | 1 | 1 | 1 | 1 |
| 1x10^−2^ | 1 | 1 | 1 | 1 |
| 5x10^−3^ | 1 | 2 | 2 | 1 |
| 1x10^−3^ | 1 | 4 | 5 | 5 |
| 5x10^−4^ | 2 | 7 | 7 | 5 |
| 1x10^−4^ | 3 | 13 | 22 | 16 |
| 5x10^−5^ | 4 | 22 | 36 | 26 |
| 1x10^−5^ | 5 | 41 | 67 | 73 |
| 5x10^−6^ | 8 | 61 | 100 | 110 |
| 1x10^−6^ | 9 | 74 | 120 | 132 |

Table S7 Fold difference of the fraction of epilepsy-eQTL SNPs that are disease-associated relative to the fraction of all SNPs that are disease-associated. FDR = false discovery rate

| FS GWAS  p-value | Epilepsy-eQTL FDR | | | |
| --- | --- | --- | --- | --- |
|  | <0.5 | <0.1 | <0.05 | <0.01 |
| 1 | 1 | 1 | 1 | 1 |
| 5x10^−1^ | 1 | 1 | 1 | 1 |
| 1x10^−1^ | 1 | 1 | 1 | 1 |
| 5x10^−2^ | 1 | 1 | 1 | 1 |
| 1x10^−2^ | 1 | 1 | 2 | 2 |
| 5x10^−3^ | 1 | 1 | 2 | 2 |
| 1x10^−3^ | 1 | 2 | 4 | 4 |
| 5x10^−4^ | 1 | 5 | 7 | 7 |
| 1x10^−4^ | 2 | 13 | 21 | 11 |
| 5x10^−5^ | 3 | 22 | 37 | 20 |
| 1x10^−5^ | 5 | 40 | 66 | 49 |
| 5x10^−6^ | 6 | 50 | 83 | 61 |
| 1x10^−6^ | 6 | 50 | 83 | 92 |

**Table S8.** Mendelian epilepsy syndromes: Mendelian disorders in which epilepsy or seizures are a cardinal feature. Data from the Online Mendelian Inheritance in Man (OMIM) database.

| Gene | Syndrome |
| --- | --- |
| AARS | Epileptic encephalopathy, early infantile, familial adult, 2 |
| ADGRV1 | Febrile seizures, familial, 29 |
| ADRA2B | Epilepsy, myoclonic, 4, |
| ALDH7A1 | Epilepsy, pyridoxine-dependent |
| ALG13 | Epileptic encephalopathy, early infantile, 36 |
| ARHGEF9 | Epileptic encephalopathy, early infantile, 8 |
| ARV1 | Epileptic encephalopathy, early infantile, 38 |
| ARX | Epileptic encephalopathy, early infantile, 1, |
| ASAH1 | Spinal muscular atrophy with progressive myoclonic epilepsy |
| BFIS1 | Seizures, benign familial infantile, 1 |
| BFIS4 | Seizures, benign familial infantile, 4 |
| BRAT1 | Rigidity and multifocal seizure syndrome, lethal neonatal |
| CACNA1D | Primary aldosteronism, seizures, and neurologic abnormalities |
| CACNA1H | Epilepsy, childhood absence, susceptibility to, 6 |
| CACNA1H | Epilepsy, idiopathic generalized, susceptibility to, 6 |
| CACNB4 | Epilepsy, juvenile myoclonic, susceptibility to, 6 |
| CACNB4 | Epilepsy, idiopathic generalized, susceptibility to, 9 |
| CASR | Epilepsy idiopathic generalized, susceptibility to, 8 |
| CDKL5 | Epileptic encephalopathy, early infantile, 2 |
| CERS1 | Epilepsy, progressive myoclonic, 8 |
| CHD2 | Epileptic encephalopathy, childhood-onset |
| CHRNA2 | Epilepsy, nocturnal frontal lobe, type 4 |
| CHRNA4 | Epilepsy, nocturnal frontal lobe, 1 |
| CHRNB2 | Epilepsy, nocturnal frontal lobe, 3 |
| CLCN2 | Epilepsy, idiopathic generalized, susceptibility to, 11 |
| CLCN2 | Epilepsy, juvenile absence, susceptibility to, 2 |
| CLCN2 | Epilepsy, juvenile myoclonic, susceptibility to, 8 |
| CLN8 | Ceroid lipofuscinosis, neuronal, 8, Northern epilepsy variant |
| CNNM2 | Hypomagnesemia, seizures, and mental retardation |
| CNTN2 | Epilepsy, myoclonic, familial adult, 5 |
| CNTNAP2 | Cortical dysplasia-focal epilepsy syndrome |
| CPA6 | Epilepsy, familial temporal lobe, 5 |
| CPA6 | Febrile seizures, familial, 11 |
| CSTB | Epilepsy, progressive myoclonic 1A |
| DEPDC5 | Epilepsy, familial focal, with variable foci |
| DIAPH1 | Seizures, cortical blindness, microcephaly syndrome |
| DNM1 | Epileptic encephalopathy, early infantile, 31 |
| DOCK7 | Epileptic encephalopathy, early infantile, 23 |
| ECA1 | Epilepsy, childhood absence, 1 |
| ECT | Centrotemporal epilepsy |
| EEF1A2 | Epileptic encephalopathy, early infantile, 33 |
| EFHC1 | Epilepsy, juvenile absence, susceptibility to, 1 |
| EFHC1 | Myoclonic epilepsy, juvenile, susceptibility to, 1 |
| EIG2 | Epilepsy, idiopathic generalized, susceptibility to, 2 |
| EIG3 | Epilepsy, idiopathic generalized, susceptibility to, 3 |
| EIG4 | Epilepsy, idiopathic generalized, susceptibility to, 4 |
| EIG5 | Epilepsy, idiopathic generalized, susceptibility to, 5 |
| EJM3 | Epilepsy, juvenile myoclonic 3 |
| EJM4 | Myoclonic epilepsy, juvenile, 4 |
| EJM9 | Epilepsy, juvenile myoclonic, susceptibility to, 9 |
| ENFL2 | Epilepsy, nocturnal frontal lobe, type 2 |
| EPM2A | Epilepsy, progressive myoclonic 2A |
| EPPS | Epilepsy, partial, with pericentral spikes |
| EPRPDC | Epilepsy, rolandic, with paroxysmal exercise-induced dystonia and writer's cramp |
| ETL2 | Epilepsy, familial temporal lobe, 2 |
| ETL4 | Epilepsy, familial temporal lobe, 4 |
| ETL6 | Epilepsy, familial temporal lobe, 6 |
| EXT2 | Seizures, scoliosis, and macrocephaly syndrome |
| FAME3 | Epilepsy, myoclonic, familial adult, 3 |
| FAME4 | Epilepsy, myoclonic, familial adult, 4 |
| FEB1 | Febrile seizures, familial, 1 |
| FEB10 | Febrile seizures, familial, 10 |
| FEB2 | Febrile seizures, familial, 2 |
| FEB5 | Febrile seizures, familial, 5 |
| FEB6 | Febrile seizures, familial, 6 |
| FEB7 | Febrile seizures, familial, 7 |
| FEB9 | Febrile seizures, familial, 9 |
| FRRS1L | Epileptic encephalopathy, early infantile, 37 |
| GABRA1 | Epilepsy, childhood absence, susceptibility to, 4 |
| GABRA1 | Epilepsy, juvenile myoclonic, susceptibility to, 5 |
| GABRA1 | Epileptic encephalopathy, early infantile, 19 |
| GABRB3 | Epilepsy, childhood absence, susceptibility to, 5 |
| GABRD | Epilepsy, generalized, with febrile seizures plus, type 5, susceptibility to |
| GABRD | Epilepsy, idiopathic generalized, 10 |
| GABRD | Epilepsy, juvenile myoclonic, susceptibility to |
| GABRG2 | Epilepsy, childhood absence, susceptibility to, 2 |
| GABRG2 | Epilepsy, generalized, with febrile seizures plus, type 3 |
| GABRG2 | Febrile seizures, familial, 8 |
| GAL | Epilepsy, familial temporal lobe, 8 |
| GEFSP4 | Epilepsy, generalized, with febrile seizures plus, type 4 |
| GEFSP6 | Epilepsy, generalized, with febrile seizures plus, type 6 |
| GEFSP8 | Epilespy, generalized, with febrile seizures plus, type 8 |
| GNAO1 | Epileptic encephalopathy, early infantile, 17 |
| GOSR2 | Epilepsy, progressive myoclonic 6 |
| GRIN2A | Epilepsy, focal, with speech disorder and with or without mental retardation |
| GRIN2B | Epileptic encephalopathy, early infantile, 27 |
| HACE1 | Spastic paraplegia and psychomotor retardation with or without seizures |
| HCN1 | Epileptic encephalopathy, early infantile, 24 |
| HWE1 | Epilepsy, hot water, 1 |
| HWE2 | Epilepsy, hot water, 2 |
| IER3IP1 | Microcephaly, epilepsy, and diabetes syndrome |
| ITPA | Epileptic encephalopathy, early infantile, 35 |
| KCNA2 | Epileptic encephalopathy, early infantile, 32 |
| KCNB1 | Epileptic encephalopathy, early infantile, 26 |
| KCNC1 | Epilepsy, progressive myoclonic 7 |
| KCNMA1 | Generalized epilepsy and paroxysmal dyskinesia |
| KCNQ2 | Epileptic encephalopathy, early infantile, 7 |
| KCNQ2 | Seizures, benign neonatal, 1 |
| KCNQ3 | Seizures, benign neonatal, type 2 |
| KCNT1 | Epilepsy, nocturnal frontal lobe, 5 |
| KCNT1 | Epileptic encephalopathy, early infantile, 14 |
| KCTD7 | Epilepsy, progressive myoclonic 3, with or without intracellular inclusions |
| LGI1 | Epilepsy, familial temporal lobe, 1 |
| LIAS | Hyperglycinemia, lactic acidosis, and seizures |
| LMNB2 | Epilepsy, progressive myoclonic, 9 |
| MED17 | Microcephaly, postnatal progressive, with seizures and brain atrophy |
| MEF2C | Mental retardation, stereotypic movements, epilepsy, and/or cerebral malformations |
| NECAP1 | Epileptic encephalopathy, early infantile, 21 |
| NHLRC1 | Epilepsy, progressive myoclonic 2B |
| PCDH19 | Epileptic encephalopathy, early infantile, 9 |
| PIGA | Multiple congenital anomalies-hypotonia-seizures syndrome 2 |
| PIGN | Multiple congenital anomalies-hypotonia-seizures syndrome 1 |
| PIGT | Multiple congenital anomalies-hypotonia-seizures syndrome 3 |
| PLCB1 | Epileptic encephalopathy, early infantile, 12 |
| PNKP | Microcephaly, seizures, and developmental delay |
| PRDM8 | Epilepsy, progressive myoclonic, 10 |
| PRICKLE1 | Epilepsy, progressive myoclonic 1B |
| PRRT2 | Seizures, benign familial infantile, 2 |
| QARS | Microcephaly, progressive, seizures, and cerebral and cerebellar atrophy |
| RELN | Epilepsy, familial temporal lobe, 7 |
| RTN4IP1 | Optic atrophy 10 with or without ataxia, mental retardation, and seizures |
| RTTN | Microcephaly, short stature, and polymicrogyria with seizures |
| SCARB2 | Epilepsy, progressive myoclonic 4, with or without renal failure |
| SCN1A | Epilepsy, generalized, with febrile seizures plus, type 2 |
| SCN1A | Febrile seizures, familial, 3A |
| SCN1B | Epilepsy, generalized, with febrile seizures plus, type 1 |
| SCN2A | Epileptic encephalopathy, early infantile, 11 |
| SCN2A | Seizures, benign familial infantile, 3 |
| SCN8A | Epileptic encephalopathy, early infantile, 13 |
| SCN9A | Epilepsy, generalized, with febrile seizures plus, type 7 |
| SCN9A | Febrile seizures, familial, 3B, |
| SIK1 | Epileptic encephalopathy, early infantile, 30 |
| SLC12A5 | Epilepsy, idiopathic generalized, susceptibility to, 14 |
| SLC12A5 | Epileptic encephalopathy, early infantile, 34 |
| SLC13A5 | Epileptic encephalopathy, early infantile, 25 |
| SLC25A12 | Epileptic encephalopathy, early infantile, 39 |
| SLC25A22 | Epileptic encephalopathy, early infantile, 3 |
| SLC2A1 | Epilepsy, idiopathic generalized, susceptibility to, 12 |
| SLC35A3 | Arthrogryposis, mental retardation, and seizures |
| SLC6A1 | Myoclonic-atonic epilepsy |
| SNIP1 | Psychomotor retardation, epilepsy, and craniofacial dysmorphism |
| SPATA5 | Epilepsy, hearing loss, and mental retardation syndrome |
| SPTAN1 | Epileptic encephalopathy, early infantile, 5 |
| SRPX2 | Rolandic epilepsy, mental retardation, and speech dyspraxia |
| ST3GAL3 | Epileptic encephalopathy, early infantile, 15 |
| STRADA | Polyhydramnios, megalencephaly, and symptomatic epilepsy |
| STX1B | Generalized epilepsy with febrile seizures plus, type 9 |
| STXBP1 | Epileptic encephalopathy, early infantile, 4 |
| SYN1 | Epilepsy, X-linked, with variable learning disabilities and behavior disorders |
| SZT2 | Epileptic encephalopathy, early infantile, 18 |
| TBC1D24 | Epileptic encephalopathy, early infantile, 16 |
| TBC1D24 | Myoclonic epilepsy, infantile, familial |
| WWOX | Epileptic encephalopathy, early infantile, 28 |

**Table S9.** SNPs that achieved at least nominal level of significance (p<0.05) in epilepsy GWAS meta-analysis and were significant (FDR<0.05) epilepsy-eQTLs or in strong LD (r^2^ = 0.8) with significant epilepsy-eQTLs, and their respective cis-genes. Focal, GGE and All refers to the focal, genetic generalised epilepsy, and all-epilepsy combined GWAS meta-analysis cohort, respectively ([6](#_ENREF_6)). Where multiple disease-risk SNPs are linked to a gene, the most significant SNP is shown, for each cohort.

| Disease-risk SNP | Significance of association with disease (p) | Study | cis-SNP | cis-gene (symbol) | cis-gene (Entrez) |
| --- | --- | --- | --- | --- | --- |
| rs35476054 | 3.988e-09 | All | rs12992163 | *TTC21B* | 100506124 |
| rs11520481 | 1.151e-06 | Focal | rs1529648 | *TTC21B* | 100506124 |
| rs2072305 | 4.976e-05 | GGE | rs11666848 | *AP3D1* | 8943 |
| rs6119530 | 5.746e-05 | All | rs56244533 | *GGT7* | 2686 |
| rs1107553 | 8.986e-05 | All | rs3985938 | *CYP2D6* | 1565 |
| rs3791843 | 9.349e-05 | GGE | rs2304002 | *TTC21B* | 100506124 |
| rs6088624 | 9.894e-05 | GGE | rs56244533 | *GGT7* | 2686 |
| rs79384878 | 0.0001123 | GGE | rs41267357 | *DNAH14* | 127602 |
| rs5758609 | 0.0001171 | Focal | rs3985938 | *CYP2D6* | 1565 |
| rs3992 | 0.0001507 | GGE | rs1543748 | *PIGP* | 51227 |
| rs1610621 | 0.0002729 | All | rs3130251 | *ZFP57* | 346171 |
| rs9271722 | 0.000301 | All | rs28366294 | *HLA-DRB1* | 3123 |
| rs376831 | 0.000675 | All | rs6897470 | *LOC100506548* | 100506548 |
| rs201858 | 0.0006752 | GGE | rs201875 | *CCDC73* | 493860 |
| rs9271722 | 0.0006762 | Focal | rs28366294 | *HLA-DRB1* | 3123 |
| rs13294415 | 0.0008346 | All | rs13294415 | *NMRK1* | 54981 |
| rs6489400 | 0.0009011 | All | rs2907509 | *FKBP4* | 2288 |
| rs112209349 | 0.0009152 | GGE | rs6988452 | *EIF3H* | 8667 |
| rs262970 | 0.001143 | All | rs262972 | *YEATS2* | 55689 |
| rs6743012 | 0.001219 | Focal | rs6743012 | *WDSUB1* | 151525 |
| rs10766478 | 0.001386 | All | rs3809096 | *LDHC* | 3948 |
| rs13100173 | 0.001537 | GGE | rs13100173 | *HYAL3* | 8372 |
| rs12581416 | 0.001541 | Focal | rs4623941 | *GLIPR1L2* | 144321 |
| rs2517467 | 0.001566 | Focal | rs2249464 | *VARS2* | 57176 |
| rs10766478 | 0.001731 | Focal | rs3809096 | *LDHC* | 3948 |
| rs7486473 | 0.001736 | All | rs73191008 | *GLIPR1L2* | 144321 |
| rs9271737 | 0.001768 | All | rs9271586 | *HLA-DQA1* | 3117 |
| rs13078629 | 0.001904 | Focal | rs3924868 | *RPL22L1* | 200916 |
| rs6573769 | 0.001907 | Focal | rs2296562 | *PLEK2* | 26499 |
| rs4821714 | 0.002073 | GGE | rs4821714 | *H1F0* | 3005 |
| rs34335167 | 0.002208 | GGE | rs12155758 | *LY6K* | 54742 |
| rs6573769 | 0.002424 | All | rs2296562 | *PLEK2* | 26499 |
| rs9272416 | 0.002894 | Focal | rs1130034 | *HLA-DQA1* | 3117 |
| rs13078629 | 0.002902 | All | rs3924868 | *RPL22L1* | 200916 |
| rs13385191 | 0.002981 | Focal | rs13394027 | *LDAH* | 60526 |
| rs1552172 | 0.003024 | Focal | rs9728345 | *NUDT17* | 200035 |
| rs7631310 | 0.003073 | GGE | rs7653826 | *GFM1* | 85476 |
| rs12868462 | 0.00308 | GGE | rs12873099 | *CCDC122* | 160857 |
| rs7172083 | 0.003643 | All | rs7172083 | *TRIM69* | 140691 |
| rs2152876 | 0.003891 | GGE | rs6919397 | *CENPW* | 387103 |
| rs2050817 | 0.003909 | GGE | rs1316588 | *NKAIN1* | 79570 |
| rs7488246 | 0.003922 | All | rs7955450 | *AMDHD1* | 144193 |
| rs10883845 | 0.003998 | All | rs11191438 | *AS3MT* | 57412 |
| rs1147137 | 0.004042 | All | rs12902649 | *TNFAIP8L3* | 388121 |
| rs559217 | 0.004067 | GGE | rs515718 | *ANKRD42* | 338699 |
| rs12609834 | 0.004195 | GGE | rs12609834 | *OPA3* | 80207 |
| rs12548797 | 0.004206 | All | rs12155758 | *LY6K* | 54742 |
| rs1610621 | 0.004288 | Focal | rs3130251 | *ZFP57* | 346171 |
| rs13041059 | 0.004326 | Focal | rs56244533 | *GGT7* | 2686 |
| rs12881353 | 0.004341 | Focal | rs12884463 | *CHURC1* | 91612 |
| rs61895512 | 0.004558 | All | rs2279518 | *ACER3* | 55331 |
| rs7488246 | 0.004742 | Focal | rs7955450 | *AMDHD1* | 144193 |
| rs62069929 | 0.004801 | GGE | rs17634022 | *SHPK* | 23729 |
| rs12516434 | 0.004926 | Focal | rs2406909 | *ATG10* | 83734 |
| rs2965190 | 0.005014 | GGE | rs1529745 | *GATAD2A* | 54815 |
| rs2119028 | 0.005096 | GGE | rs2298746 | *XRRA1* | 143570 |
| rs137075 | 0.005249 | Focal | rs137112 | *CYB5R3* | 1727 |
| rs2488808 | 0.005283 | All | rs1018789 | *GRK4* | 2868 |
| rs6658381 | 0.005346 | All | rs11587667 | *STPG1* | 90529 |
| rs77997984 | 0.005467 | GGE | rs4586394 | *UBE2Q2L* | 100505679 |
| rs6771080 | 0.005487 | Focal | rs1877960 | *LARS2* | 23395 |
| rs12722743 | 0.005692 | All | rs12756688 | *SHISA4* | 149345 |
| rs15810 | 0.005928 | GGE | rs2463830 | *ASRGL1* | 80150 |
| rs10182643 | 0.005938 | All | rs13394027 | *LDAH* | 60526 |
| rs6658381 | 0.006204 | Focal | rs11587667 | *STPG1* | 90529 |
| rs6924799 | 0.006288 | Focal | rs67699103 | *MCUR1* | 63933 |
| rs3095151 | 0.006556 | All | rs4678 | *VARS2* | 57176 |
| rs2075212 | 0.006856 | GGE | rs2075212 | *SEMA4F* | 10505 |
| rs10793223 | 0.007205 | GGE | rs7119361 | *ACER3* | 55331 |
| rs890502 | 0.00736 | Focal | rs11636960 | *SPTBN5* | 51332 |
| rs17387775 | 0.007398 | Focal | rs900835 | *SLC2A1-AS1* | 440584 |
| rs10887265 | 0.007423 | All | rs1042454 | *RGR* | 5995 |
| rs825175 | 0.007433 | Focal | rs825175 | *UBE2U* | 148581 |
| rs4413573 | 0.007439 | GGE | rs702399 | *WDR55* | 54853 |
| rs755447 | 0.007563 | All | rs77386309 | *SLC26A1* | 10861 |
| rs2714869 | 0.007585 | GGE | rs2714878 | *SNX13* | 23161 |
| rs1704392 | 0.007888 | All | rs11636960 | *SPTBN5* | 51332 |
| rs736141 | 0.007967 | Focal | rs1042454 | *RGR* | 5995 |
| rs1130165 | 0.007993 | All | rs12620727 | *ADI1* | 55256 |
| rs911737 | 0.00837 | Focal | rs773144 | *NMRK1* | 54981 |
| rs28666643 | 0.008578 | Focal | rs1543748 | *PIGP* | 51227 |
| rs35117964 | 0.008635 | GGE | rs35265698 | *HLA-DRB1* | 3123 |
| rs262969 | 0.009028 | Focal | rs262972 | *YEATS2* | 55689 |
| rs6462901 | 0.009028 | GGE | rs11514715 | *POU6F2* | 11281 |
| rs7516301 | 0.009086 | GGE | rs9425311 | *DNM3* | 26052 |
| rs9929108 | 0.009376 | All | rs7184944 | *BCAR1* | 9564 |
| rs113301808 | 0.009549 | Focal | rs11965435 | *TRERF1* | 55809 |
| rs10891118 | 0.009764 | All | rs111971680 | *FDX1* | 2230 |
| rs34237273 | 0.00977 | GGE | rs2228410 | *AOAH* | 313 |
| rs137075 | 0.009808 | All | rs137112 | *CYB5R3* | 1727 |
| rs4292733 | 0.009999 | GGE | rs4292733 | *ARHGEF10* | 9639 |
| rs62321860 | 0.01001 | All | rs3762839 | *TRPC3* | 7222 |
| rs12718910 | 0.01019 | Focal | rs11764792 | *FIGNL1* | 63979 |
| rs113912503 | 0.01094 | GGE | rs12443600 | *OGFOD1* | 55239 |
| rs7172083 | 0.01103 | Focal | rs7172083 | *TRIM69* | 140691 |
| rs1147137 | 0.01117 | Focal | rs12902649 | *TNFAIP8L3* | 388121 |
| rs1556581 | 0.01129 | GGE | rs3120047 | *TIE1* | 7075 |
| rs35800067 | 0.0114 | GGE | rs6443264 | *TADA3* | 10474 |
| rs141166762 | 0.01142 | GGE | rs77386309 | *SLC26A1* | 10861 |
| rs17460595 | 0.01144 | All | rs2035512 | *FRAS1* | 80144 |
| rs111884657 | 0.01162 | All | rs79414850 | *DNAJC15* | 29103 |
| rs11622591 | 0.01172 | All | rs76541382 | *ZBTB1* | 22890 |
| rs11263770 | 0.01175 | All | rs4796224 | *MYO19* | 80179 |
| rs755447 | 0.01176 | Focal | rs77386309 | *SLC26A1* | 10861 |
| rs11144225 | 0.01192 | GGE | rs11144225 | *NMRK1* | 54981 |
| rs6804230 | 0.01235 | GGE | rs62288301 | *LXN* | 56925 |
| rs7235202 | 0.01249 | All | rs8088313 | *CEP192* | 55125 |
| rs10899293 | 0.01273 | Focal | rs2279518 | *ACER3* | 55331 |
| rs7631372 | 0.01284 | GGE | rs62246275 | *THUMPD3* | 25917 |
| rs3741032 | 0.01312 | GGE | rs10840448 | *MRVI1* | 10335 |
| rs4455713 | 0.01321 | Focal | rs6914191 | *HTATSF1P2* | 401233 |
| rs3736922 | 0.01342 | Focal | rs11191438 | *AS3MT* | 57412 |
| rs12904319 | 0.0136 | All | rs11856606 | *SNX33* | 257364 |
| rs67059212 | 0.01362 | All | rs9462853 | *GNMT* | 27232 |
| rs4666483 | 0.01363 | All | rs4666483 | *OSR1* | 130497 |
| rs2844762 | 0.0137 | GGE | rs2844762 | *HCG18* | 414777 |
| rs11514715 | 0.01375 | All | rs11514715 | *POU6F2* | 11281 |
| rs11622298 | 0.01409 | All | rs1957420 | *CHURC1* | 91612 |
| rs11086166 | 0.01415 | Focal | rs6062949 | *EEF1A2* | 1917 |
| rs11086166 | 0.01415 | Focal | rs62208042 | *KCNQ2* | 3785 |
| rs1130165 | 0.01428 | Focal | rs12620727 | *ADI1* | 55256 |
| rs11843884 | 0.01434 | All | rs11840986 | *TDRD3* | 81550 |
| rs4924 | 0.01447 | All | rs2432537 | *AMFR* | 267 |
| rs2786691 | 0.01467 | GGE | rs10927360 | *EFCAB2* | 84288 |
| rs2377056 | 0.01472 | All | rs364343 | *RPL13* | 6137 |
| rs2377056 | 0.01472 | All | rs57775530 | *SPG7* | 6687 |
| rs112172758 | 0.01491 | All | rs41267357 | *DNAH14* | 127602 |
| rs3749482 | 0.01502 | Focal | rs3811747 | *FRAS1* | 80144 |
| rs3801032 | 0.01523 | All | rs4724768 | *EIF2AK1* | 27102 |
| rs10750454 | 0.01559 | GGE | rs10894279 | *SNX19* | 399979 |
| rs6771080 | 0.01607 | All | rs1877960 | *LARS2* | 23395 |
| rs4576787 | 0.01609 | Focal | rs111971680 | *FDX1* | 2230 |
| rs11264505 | 0.01644 | GGE | rs6688600 | *APOA1BP* | 128240 |
| rs12624148 | 0.01674 | GGE | rs6547735 | *GPN1* | 11321 |
| rs78870788 | 0.01681 | Focal | rs77811147 | *UFSP2* | 55325 |
| rs6857766 | 0.01688 | Focal | rs6857766 | *ANXA5* | 308 |
| rs832552 | 0.01688 | GGE | rs252901 | *SETD9* | 133383 |
| rs3745826 | 0.0169 | GGE | rs7250817 | *ZNF667* | 63934 |
| rs376831 | 0.01711 | Focal | rs6897470 | *LOC100506548* | 100506548 |
| rs4855617 | 0.01721 | GGE | rs9879947 | *CD47* | 961 |
| rs9840626 | 0.01723 | GGE | rs9840626 | *CCDC174* | 51244 |
| rs2296968 | 0.01733 | Focal | rs2296968 | *SOHLH2* | 54937 |
| rs1108902 | 0.01736 | All | rs900835 | *SLC2A1-AS1* | 440584 |
| rs912927 | 0.01746 | All | rs9546759 | *CCDC169* | 728591 |
| rs912927 | 0.01746 | All | rs9546759 | *SOHLH2* | 54937 |
| rs350853 | 0.01754 | GGE | rs350854 | *SIRT6* | 51548 |
| rs1074347 | 0.01762 | GGE | rs1074347 | *ANKRD12* | 23253 |
| rs138762909 | 0.01766 | GGE | rs74463044 | *CCDC171* | 203238 |
| rs912927 | 0.01769 | Focal | rs9546759 | *CCDC169* | 728591 |
| rs45441993 | 0.01775 | GGE | rs3985938 | *CYP2D6* | 1565 |
| rs1678599 | 0.01796 | GGE | rs1629816 | *SEC13* | 6396 |
| rs7130160 | 0.01832 | Focal | rs75115775 | *SIDT2* | 51092 |
| rs11760442 | 0.01833 | Focal | rs752637 | *IRF5* | 3663 |
| rs3087633 | 0.01847 | All | rs113625841 | *FAM229B* | 619208 |
| rs140264 | 0.01877 | Focal | rs140264 | *DDT* | 1652 |
| rs140264 | 0.01877 | Focal | rs140264 | *DDTL* | 100037417 |
| rs140264 | 0.01877 | Focal | rs140264 | *GSTT2B* | 653689 |
| rs12538339 | 0.01884 | GGE | rs4724768 | *EIF2AK1* | 27102 |
| rs918727 | 0.01901 | All | rs918727 | *MTHFSD* | 64779 |
| rs7533521 | 0.01904 | GGE | rs2767306 | *ARPC5* | 10092 |
| rs4821714 | 0.01921 | All | rs4821714 | *H1F0* | 3005 |
| rs2159983 | 0.01924 | All | rs350854 | *SIRT6* | 51548 |
| rs11234092 | 0.01951 | GGE | rs11234074 | *KRTAP5-8* | 57830 |
| rs12873099 | 0.01992 | All | rs12873099 | *CCDC122* | 160857 |
| rs11760442 | 0.02023 | All | rs752637 | *IRF5* | 3663 |
| rs1693556 | 0.02053 | All | rs1786344 | *SNX31* | 169166 |
| rs8009022 | 0.02087 | GGE | rs114801506 | *DHRS4L2* | 317749 |
| rs2853923 | 0.02159 | Focal | rs2524058 | *HLA-C* | 3107 |
| rs11086166 | 0.02244 | All | rs6062949 | *EEF1A2* | 1917 |
| rs11086166 | 0.02244 | All | rs62208042 | *KCNQ2* | 3785 |
| rs7782527 | 0.02313 | GGE | rs71563153 | *TYW1* | 55253 |
| rs66570840 | 0.0233 | All | rs12460702 | *ZNF264* | 9422 |
| rs7488246 | 0.02406 | GGE | rs7955450 | *AMDHD1* | 144193 |
| rs12612925 | 0.02432 | GGE | rs13021969 | *SNRNP27* | 11017 |
| rs7724201 | 0.02478 | GGE | rs381471 | *LOC100506548* | 100506548 |
| rs747632 | 0.02481 | All | rs4729431 | *FLJ30064* | 644975 |
| rs269344 | 0.02486 | GGE | rs269327 | *WLS* | 79971 |
| rs145708598 | 0.02491 | All | rs143917969 | *STAG3L4* | 64940 |
| rs2032463 | 0.02528 | Focal | rs2488809 | *GRK4* | 2868 |
| rs56007903 | 0.02595 | Focal | rs76541382 | *ZBTB1* | 22890 |
| rs1998811 | 0.02612 | GGE | rs3818933 | *PPIL6* | 285755 |
| rs2899341 | 0.02613 | Focal | rs138342 | *XPNPEP3* | 63929 |
| rs12608562 | 0.02642 | Focal | rs12608562 | *PIAS4* | 51588 |
| rs35080624 | 0.02651 | Focal | rs139582992 | *ZNF568* | 374900 |
| rs71597205 | 0.02661 | GGE | rs2488809 | *GRK4* | 2868 |
| rs62066346 | 0.02684 | Focal | rs385301 | *AKAP10* | 11216 |
| rs4959761 | 0.02711 | GGE | rs12192697 | *HTATSF1P2* | 401233 |
| rs2870783 | 0.02712 | GGE | rs2576187 | *TMBIM4* | 51643 |
| rs11747814 | 0.02729 | All | rs17690965 | *KIF3A* | 11127 |
| rs4666483 | 0.02771 | GGE | rs4666483 | *OSR1* | 130497 |
| rs1534140 | 0.02773 | Focal | rs10017725 | *SLC25A4* | 291 |
| rs1667351 | 0.02778 | All | rs1667351 | *ZNF568* | 374900 |
| rs1480287 | 0.02782 | GGE | rs6857766 | *ANXA5* | 308 |
| rs2854195 | 0.02839 | All | rs2597634 | *FTSJ3* | 117246 |
| rs59117307 | 0.02843 | All | rs4255343 | *AXDND1* | 126859 |
| rs3213946 | 0.02847 | All | rs11682248 | *PHOSPHO2* | 493911 |
| rs11264505 | 0.02866 | All | rs6688600 | *APOA1BP* | 128240 |
| rs12608562 | 0.02898 | All | rs12608562 | *PIAS4* | 51588 |
| rs696219 | 0.0292 | All | rs1629816 | *SEC13* | 6396 |
| rs4949365 | 0.02923 | Focal | rs4949365 | *NKAIN1* | 79570 |
| rs11979371 | 0.02929 | GGE | rs116026907 | *STAG3L4* | 64940 |
| rs10822229 | 0.02943 | GGE | rs10995617 | *PRKG1* | 5592 |
| rs61854782 | 0.02967 | Focal | rs61854809 | *PBLD* | 64081 |
| rs4802201 | 0.0299 | All | rs10416434 | *ZNF404* | 342908 |
| rs144440496 | 0.02992 | All | rs114362791 | *FANCD2OS* | 115795 |
| rs2381409 | 0.03049 | Focal | rs10758326 | *TMEM8B* | 51754 |
| rs1020393 | 0.03053 | All | rs75115775 | *SIDT2* | 51092 |
| rs12443191 | 0.03089 | Focal | rs1869302 | *MRPL42P5* | 359821 |
| rs78870788 | 0.03154 | All | rs77811147 | *UFSP2* | 55325 |
| rs10903016 | 0.03168 | Focal | rs4984874 | *DECR2* | 26063 |
| rs2683056 | 0.03178 | Focal | rs2683047 | *CYP4F3* | 4051 |
| rs4729431 | 0.0318 | Focal | rs4729431 | *FLJ30064* | 644975 |
| rs28362345 | 0.03218 | GGE | rs9263871 | *HCG27* | 253018 |
| rs11932526 | 0.03239 | GGE | rs2301717 | *PLA2G12A* | 81579 |
| rs7618141 | 0.0326 | GGE | rs62263868 | *PARP15* | 165631 |
| rs8087334 | 0.03291 | All | rs1074347 | *ANKRD12* | 23253 |
| rs5760108 | 0.03356 | All | rs140264 | *DDT* | 1652 |
| rs5760108 | 0.03356 | All | rs140264 | *DDTL* | 100037417 |
| rs5760108 | 0.03356 | All | rs140264 | *GSTT2B* | 653689 |
| rs9929108 | 0.03362 | GGE | rs7184944 | *BCAR1* | 9564 |
| rs2362450 | 0.03365 | Focal | rs9876781 | *TREX1* | 11277 |
| rs17203570 | 0.03428 | GGE | rs9271586 | *HLA-DQA1* | 3117 |
| rs2482038 | 0.03429 | Focal | rs6602619 | *CAMK1D* | 57118 |
| rs2018027 | 0.03432 | GGE | rs9728345 | *NUDT17* | 200035 |
| rs4715562 | 0.03433 | All | rs67699103 | *MCUR1* | 63933 |
| rs825166 | 0.03443 | All | rs825183 | *UBE2U* | 148581 |
| rs2854195 | 0.03448 | Focal | rs2597634 | *FTSJ3* | 117246 |
| rs7754722 | 0.03458 | GGE | rs7754722 | *ALDH8A1* | 64577 |
| rs3825798 | 0.03459 | GGE | rs12902649 | *TNFAIP8L3* | 388121 |
| rs10207536 | 0.03462 | All | rs13009889 | *ORMDL1* | 94101 |
| rs2380793 | 0.03463 | Focal | rs57790078 | *LRP1B* | 53353 |
| rs11263775 | 0.0349 | GGE | rs1061121 | *DHRS11* | 79154 |
| rs11124962 | 0.03497 | Focal | rs4953036 | *LRPPRC* | 10128 |
| rs11671113 | 0.03506 | GGE | rs1051500 | *ZNF132* | 7691 |
| rs34042070 | 0.03508 | GGE | rs3794695 | *HP* | 3240 |
| rs34042070 | 0.03508 | GGE | rs3794695 | *HPR* | 3250 |
| rs8023115 | 0.03511 | All | rs1257265 | *SETD3* | 84193 |
| rs12666932 | 0.03514 | Focal | rs10260959 | *SSPO* | 23145 |
| rs6544728 | 0.03516 | All | rs4952692 | *LRPPRC* | 10128 |
| rs4901318 | 0.03527 | GGE | rs4901316 | *FERMT2* | 10979 |
| rs71502657 | 0.03575 | GGE | rs8635 | *RMDN1* | 51115 |
| rs8102502 | 0.0358 | Focal | rs1529745 | *GATAD2A* | 54815 |
| rs11670750 | 0.03618 | GGE | rs11670750 | *CARD8* | 22900 |
| rs191865 | 0.03622 | GGE | rs397969 | *AKAP10* | 11216 |
| rs11263770 | 0.03626 | Focal | rs4796224 | *MYO19* | 80179 |
| rs75887719 | 0.03637 | All | rs11965435 | *TRERF1* | 55809 |
| rs66471312 | 0.03641 | Focal | rs67613346 | *FGL2* | 10875 |
| rs1808723 | 0.0367 | All | rs1808723 | *LYSMD4* | 145748 |
| rs10995617 | 0.03693 | All | rs9414805 | *PRKG1* | 5592 |
| rs11747814 | 0.03742 | GGE | rs17690965 | *KIF3A* | 11127 |
| rs9888796 | 0.03777 | All | rs9888796 | *SLC7A6* | 9057 |
| rs12368421 | 0.03792 | Focal | rs186622026 | *EEA1* | 8411 |
| rs4804815 | 0.03803 | All | rs62125171 | *CLEC4GP1* | 440508 |
| rs830167 | 0.03814 | GGE | rs262972 | *YEATS2* | 55689 |
| rs11856606 | 0.03823 | Focal | rs11856606 | *SNX33* | 257364 |
| rs2270421 | 0.03855 | Focal | rs2287395 | *GSTZ1* | 2954 |
| rs11587553 | 0.03868 | All | rs11805687 | *NKAIN1* | 79570 |
| rs7934248 | 0.03977 | GGE | rs7950145 | *FDX1* | 2230 |
| rs11584518 | 0.03983 | Focal | rs12144920 | *STIL* | 6491 |
| rs66471312 | 0.04001 | All | rs67613346 | *FGL2* | 10875 |
| rs373628328 | 0.04002 | Focal | rs143917969 | *STAG3L4* | 64940 |
| rs11941935 | 0.04005 | Focal | rs6854452 | *LIAS* | 11019 |
| rs75706874 | 0.04047 | Focal | rs139975905 | *KIAA1456* | 57604 |
| rs9462853 | 0.04082 | Focal | rs9462853 | *GNMT* | 27232 |
| rs10887265 | 0.04121 | GGE | rs1042454 | *RGR* | 5995 |
| rs1011052 | 0.04126 | All | rs6841872 | *USP53* | 54532 |
| rs11520127 | 0.04129 | Focal | rs11174568 | *PPM1H* | 57460 |
| rs2130603 | 0.04132 | All | rs6919397 | *CENPW* | 387103 |
| rs4802201 | 0.04134 | Focal | rs10416434 | *ZNF404* | 342908 |
| rs147629910 | 0.04176 | GGE | rs2404734 | *SCAPER* | 49855 |
| rs2915816 | 0.0418 | Focal | rs2964585 | *G3BP1* | 10146 |
| rs75743201 | 0.04193 | GGE | rs11965435 | *TRERF1* | 55809 |
| rs1290618 | 0.04212 | GGE | rs2072604 | *CYP4F3* | 4051 |
| rs79946974 | 0.04235 | GGE | rs11840986 | *TDRD3* | 81550 |
| rs696219 | 0.04245 | Focal | rs1629816 | *SEC13* | 6396 |
| rs953978 | 0.04266 | GGE | rs9788649 | *LACTB* | 114294 |
| rs344356 | 0.0427 | All | rs1623559 | *FAHD1* | 81889 |
| rs918727 | 0.04282 | GGE | rs918727 | *MTHFSD* | 64779 |
| rs12892702 | 0.04297 | Focal | rs12890364 | *LINC00341* | 79686 |
| rs7639743 | 0.04348 | All | rs9876781 | *TREX1* | 11277 |
| rs4757659 | 0.04355 | GGE | rs4757659 | *LDHC* | 3948 |
| rs9729480 | 0.04404 | All | rs9728345 | *NUDT17* | 200035 |
| rs2591140 | 0.04495 | Focal | rs2591145 | *KCNJ3* | 3760 |
| rs4968831 | 0.04506 | All | rs1055448 | *ABCA6* | 23460 |
| rs6427671 | 0.04518 | Focal | rs1046379 | *S100A13* | 6284 |
| rs9654850 | 0.04526 | All | rs71563153 | *TYW1* | 55253 |
| rs8026587 | 0.04556 | Focal | rs2470167 | *DMXL2* | 23312 |
| rs2118402 | 0.04584 | All | rs3755007 | *GNLY* | 10578 |
| rs12386146 | 0.0466 | All | rs13006627 | *SLC35F5* | 80255 |
| rs6570023 | 0.0471 | Focal | rs9494332 | *LINC00271* | 100131814 |
| rs6664058 | 0.04723 | Focal | rs1174686 | *ARPC5* | 10092 |
| rs555133 | 0.04779 | All | rs238162 | *ZNFX1* | 57169 |
| rs4968831 | 0.04784 | Focal | rs1055448 | *ABCA6* | 23460 |
| rs10787577 | 0.04792 | GGE | rs11591491 | *ATRNL1* | 26033 |
| rs62016914 | 0.04818 | All | rs720251 | *UACA* | 55075 |
| rs13405290 | 0.04847 | GGE | rs56216206 | *ANO7* | 50636 |
| rs8086346 | 0.04861 | GGE | rs8088313 | *CEP192* | 55125 |
| rs9900214 | 0.04878 | GGE | rs4523980 | *RNF213* | 57674 |
| rs12529490 | 0.04888 | Focal | rs1748237 | *SUPT3H* | 8464 |
| rs6429094 | 0.04899 | GGE | rs7412979 | *TBCE* | 6905 |
| rs6988452 | 0.04903 | All | rs6988452 | *EIF3H* | 8667 |
| rs2118402 | 0.04911 | GGE | rs3755007 | *GNLY* | 10578 |
| rs2731112 | 0.04925 | All | rs2566335 | *GFM1* | 85476 |
| rs12100924 | 0.04938 | Focal | rs1257267 | *SETD3* | 84193 |
| rs8009022 | 0.04951 | All | rs114801506 | *DHRS4L2* | 317749 |
| rs111884657 | 0.04978 | Focal | rs79414850 | *DNAJC15* | 29103 |
| rs11147932 | 0.04996 | Focal | rs12873099 | *CCDC122* | 160857 |

**Table S10.** Results of Sherlock analysis ([7](#_ENREF_7)) of epilepsy GWAS meta-analysis ([6](#_ENREF_6)) and epilepsy eQTL data.

| Gene symbol | Log Bayes Factor | p-value |
| --- | --- | --- |
| *TTC21B* | 5.44977 | 1.74E-05 |
| *lincRNA:chr5:82323780-82326924_R* | 2.94372 | 0.001007 |
| *CYP2D6* | 2.0408 | 0.002361 |
| *ZFP57* | 2.03183 | 0.002361 |
| *GGT7* | 1.98523 | 0.002604 |
| *GLIPR1L2* | 1.59916 | 0.004306 |
| *TRIM69* | 1.57387 | 0.004375 |
| *AS3MT* | 1.46222 | 0.005 |
| *YEATS2* | 1.16992 | 0.007535 |
| *NMRK1* | 1.08459 | 0.008472 |
| *LOC100506548* | 0.779525 | 0.012292 |
| *lincRNA:chr7:30601413-30603609_R* | 0.551207 | 0.017674 |
| *ADI1* | 0.531795 | 0.018333 |
| *CYP17A1-AS1* | 0.497963 | 0.019375 |
| *C2orf43* | 0.495985 | 0.01941 |
| *lincRNA:chr7:30563982-30601116_R* | 0.485846 | 0.019757 |
| *TDRD3* | 0.418322 | 0.022222 |
| *lincRNA:chr7:30601081-30617375_R* | 0.394644 | 0.02309 |
| *OSR1* | 0.383918 | 0.023785 |
| *SNX33* | 0.339344 | 0.025382 |
| *lincRNA:chr19:41976160-41983985_R* | 0.312134 | 0.026563 |
| *SHISA4* | 0.276551 | 0.028368 |
| *FDX1* | 0.263699 | 0.029271 |
| *lincRNA:chr7:30590168-30601268_R* | 0.249913 | 0.029965 |
| *SLC2A1-AS1* | 0.242893 | 0.030417 |
| *RGR* | 0.218831 | 0.032361 |
| *lincRNA:chr18:966025-1025450_F* | 0.205037 | 0.033646 |
| *lincRNA:chr19:41962278-41985193_R* | 0.142065 | 0.039618 |
| *ZBTB1* | 0.136192 | 0.040347 |
| *SIRT6* | 0.123148 | 0.042153 |
| *PIAS4* | 0.0993351 | 0.044653 |
| *AMDHD1* | 0.0758105 | 0.048472 |
| *lincRNA:chr8:587535-591207_R* | 0.0639324 | 0.050694 |
| *TNFAIP8L3* | 0.0492287 | 0.054167 |
| *H1F0* | 0.0491263 | 0.054167 |
| *lincRNA:chr11:65190268-65193997_F* | 0.0478494 | 0.05441 |
| *lincRNA:chr7:30590171-30601128_R* | 0.0396653 | 0.056424 |
| *STPG1* | 0.0324251 | 0.058299 |
| *lincRNA:chr7:26438338-26533562_F* | 0.0179505 | 0.062708 |
| *lincRNA:chr7:26438525-26533683_F* | 0.0132804 | 0.064375 |
| *lincRNA:chr11:82783427-82804838_F* | 0.00427547 | 0.067708 |
| *lincRNA:chr6:22860365-23017049_F* | 0.00411221 | 0.067813 |
| *lincRNA:chr8:590406-605477_F* | 0.00374445 | 0.067986 |
| *CCDC122* | 0.000663474 | 0.069444 |
| *LYSMD4* | 9.41E-05 | 0.069583 |
| *FAM229B* | -0.00192696 | 0.07059 |
| *GRK4* | -0.0044272 | 0.071736 |
| *lincRNA:chr2:8708024-8721899_R* | -0.00668305 | 0.072813 |
| *TRERF1* | -0.0110908 | 0.075278 |
| *POU6F2* | -0.0119432 | 0.075938 |
| *CCDC90A* | -0.0144219 | 0.077847 |
| *SLC7A6* | -0.0183639 | 0.080764 |
| *RSPH3* | -0.0193826 | 0.081424 |
| *RNF213* | -0.0209371 | 0.082778 |
| *AXDND1* | -0.0235434 | 0.085278 |
| *lincRNA:chr9:33011575-33024766_R* | -0.0243642 | 0.085972 |
| *EIF3H* | -0.0246233 | 0.086146 |
| *PRKG1* | -0.0252143 | 0.086771 |
| *C17orf97* | -0.0295405 | 0.091806 |
| *C14orf178* | -0.0311322 | 0.093854 |
| *SLC25A4* | -0.0326635 | 0.096076 |
| *TRPC3* | -0.0345598 | 0.099479 |
| *lincRNA:chr10:88688970-88694220_F* | -0.0350008 | 0.100243 |
| *PIGP* | -0.0362315 | 0.104271 |
| *ANXA5* | -0.0365446 | 0.106215 |
| *KIAA1841* | -0.0367053 | 0.106875 |
| *lincRNA:chr1:219488677-219519402_F* | -0.0371065 | 0.109097 |
| *QSOX1* | -0.0371691 | 0.109306 |
| *MRPL27* | -0.0373043 | 0.110347 |
| *STAT4* | -0.0378698 | 0.112847 |
| *lincRNA:chr7:18274475-18527150_F* | -0.037996 | 0.113958 |
| *SEMA5A* | -0.0385041 | 0.116146 |
| *FIGNL1* | -0.0385976 | 0.117257 |
| *KCNIP1* | -0.0389065 | 0.118542 |
| *CTNNA3* | -0.0396276 | 0.122083 |
| *lincRNA:chr8:561925-598750_F* | -0.0397159 | 0.122569 |
| *lincRNA:chr7:30590243-30601332_R* | -0.0398413 | 0.12309 |
| *OR9Q2* | -0.0400441 | 0.124306 |
| *KCNJ3* | -0.040207 | 0.125625 |
| *LIPT2* | -0.0403305 | 0.126667 |
| *lincRNA:chr22:27066108-27066654_R* | -0.0404422 | 0.127882 |
| *MYO19* | -0.0404494 | 0.127951 |
| *CEP164* | -0.0405185 | 0.128542 |
| *NCKAP5* | -0.0405613 | 0.129236 |
| *STAG3L4* | -0.0405741 | 0.129375 |
| *GSTZ1* | -0.0407212 | 0.130833 |
| *SPG7* | -0.0408647 | 0.131944 |
| *SLC27A6* | -0.0409743 | 0.132674 |
| *FLJ32255* | -0.0418225 | 0.138472 |
| *SNX11* | -0.0420813 | 0.140139 |
| *FKBP4* | -0.0421135 | 0.140313 |
| *CLECL1* | -0.0421485 | 0.140729 |
| *RNASEH2B* | -0.0421532 | 0.140799 |
| *SULT1C2* | -0.0426854 | 0.146111 |
| *BHMT* | -0.0426995 | 0.146215 |
| *CCT3* | -0.0434287 | 0.151319 |
| *C7orf55* | -0.0436316 | 0.152847 |
| *lincRNA:chr2:217380530-217421230_F* | -0.0437865 | 0.153854 |
| *G3BP1* | -0.0441698 | 0.156562 |
| *DCLRE1B* | -0.0442323 | 0.157153 |
| *ERAP1* | -0.044919 | 0.162917 |
| *WLS* | -0.0452233 | 0.165868 |
| *RCVRN* | -0.0452957 | 0.166319 |
| *CAMK1D* | -0.0455222 | 0.168715 |
| *ZNF707* | -0.0460679 | 0.17309 |
| *S100A13* | -0.0462688 | 0.175174 |
| *lincRNA:chr8:66501846-66512821_R* | -0.0464151 | 0.176632 |
| *BCAR1* | -0.0466813 | 0.179097 |
| *MCOLN2* | -0.0467666 | 0.179896 |
| *lincRNA:chr2:114566880-114619605_R* | -0.0469233 | 0.180556 |
| *lincRNA:chr5:36701011-36702056_R* | -0.0469852 | 0.180833 |
| *USP53* | -0.0469927 | 0.180833 |
| *WDR55* | -0.0471062 | 0.181458 |
| *MRPS10* | -0.048168 | 0.189653 |
| *UFSP2* | -0.0482332 | 0.190208 |
| *lincRNA:chr9:248650-264187_F* | -0.0483109 | 0.190833 |
| *TAF4B* | -0.0483847 | 0.191319 |
| *lincRNA:chr1:119683902-119693077_F* | -0.0486076 | 0.193125 |
| *KCNIP4* | -0.0488648 | 0.195208 |
| *RPL13* | -0.0493722 | 0.198403 |
| *IQCA1* | -0.0496693 | 0.2 |
| *lincRNA:chr14:53687975-53843100_F* | -0.0501845 | 0.203854 |
| *SUPT3H* | -0.0505087 | 0.206111 |
| *PDGFRB* | -0.0506492 | 0.207674 |
| *TAS2R31* | -0.0507744 | 0.208299 |
| *SSR4P1* | -0.0512048 | 0.21066 |
| *LRP1B* | -0.0516471 | 0.212986 |
| *TOB2P1* | -0.0518063 | 0.213924 |
| *FRMD4A* | -0.051871 | 0.21434 |
| *WDR36* | -0.0521819 | 0.216667 |
| *TMEM150A* | -0.0521966 | 0.216736 |
| *C7orf50* | -0.0524345 | 0.218681 |
| *CUX1* | -0.0524874 | 0.218993 |
| *TMBIM4* | -0.0526678 | 0.221007 |
| *STK33* | -0.0526899 | 0.221215 |
| *LRRC7* | -0.0528657 | 0.222604 |
| *CDKL2* | -0.0533175 | 0.226146 |
| *LIAS* | -0.0535162 | 0.228125 |
| *TOR1B* | -0.0535414 | 0.228264 |
| *FSTL5* | -0.0538632 | 0.230903 |
| *PRKAA1* | -0.0540379 | 0.23184 |
| *lincRNA:chr1:204525202-204575852_R* | -0.0543982 | 0.233958 |
| *lincRNA:chr2:64455559-64478475_F* | -0.0544966 | 0.234444 |
| *HSD17B12* | -0.0547088 | 0.236354 |
| *lincRNA:chr1:93797801-93799039_R* | -0.054758 | 0.236667 |
| *TADA3* | -0.0558108 | 0.242708 |
| *ZNF132* | -0.056006 | 0.243681 |
| *PCGF3* | -0.056121 | 0.244792 |
| *LARS2* | -0.0567699 | 0.248646 |
| *SEMA4F* | -0.0568743 | 0.249271 |
| *DDX47* | -0.0570065 | 0.250139 |
| *CNOT6L* | -0.0580123 | 0.254201 |
| *KLHDC10* | -0.0582057 | 0.255069 |
| *OPA3* | -0.0582432 | 0.255382 |
| *PDE3A* | -0.0584709 | 0.25691 |
| *XLOC_000918* | -0.0592598 | 0.260174 |
| *lincRNA:chr4:6672489-6675591_R* | -0.0594469 | 0.261528 |
| *NPEPL1* | -0.0600535 | 0.265382 |
| *ATP8B3* | -0.0601087 | 0.265764 |
| *TCP11L1* | -0.0602302 | 0.266667 |
| *ZPBP* | -0.0609934 | 0.270451 |
| *BDKRB1* | -0.0611055 | 0.270937 |
| *lincRNA:chr2:152627254-152653279_R* | -0.0612193 | 0.271562 |
| *ZMIZ1* | -0.0612636 | 0.272014 |
| *PFN4* | -0.0614023 | 0.272917 |
| *lincRNA:chr20:58662941-58676395_F* | -0.0617008 | 0.275104 |
| *DYX1C1* | -0.0617115 | 0.275243 |
| *LINC00294* | -0.0620558 | 0.277431 |
| *SYCE1L* | -0.0622421 | 0.278472 |
| *MRPL34* | -0.0626773 | 0.280938 |
| *ZKSCAN7* | -0.0629346 | 0.28184 |
| *NUSAP1* | -0.0633166 | 0.283819 |
| *TMEM57* | -0.0641563 | 0.288056 |
| *NUDT17* | -0.0645638 | 0.291771 |
| *LINC00290* | -0.0651407 | 0.295382 |
| *EFHC1* | -0.0653537 | 0.29684 |
| *AMFR* | -0.0654503 | 0.297639 |
| *FAM221A* | -0.0660275 | 0.299549 |
| *LRRFIP2* | -0.0661661 | 0.300069 |
| *C10orf113* | -0.0664754 | 0.301597 |
| *lincRNA:chr21:29817053-30047175_R* | -0.0665322 | 0.301979 |
| *TNFRSF11B* | -0.0671107 | 0.304236 |
| *TYW1* | -0.0671956 | 0.304861 |
| *lincRNA:chr10:67330944-67543094_F* | -0.0680345 | 0.308021 |
| *CEP192* | -0.0681349 | 0.308403 |
| *STIL* | -0.0686922 | 0.310625 |
| *TCOF1* | -0.0688075 | 0.311215 |
| *PPHLN1* | -0.0689139 | 0.311389 |
| *DTNA* | -0.068977 | 0.311528 |
| *EXOC3* | -0.0689911 | 0.311563 |
| *CCDC171* | -0.0689943 | 0.311597 |
| *GRID2* | -0.0690311 | 0.311632 |
| *lincRNA:chr2:24355021-24385921_F* | -0.0691819 | 0.312326 |
| *CBR4* | -0.0704652 | 0.316563 |
| *AASDHPPT* | -0.0706282 | 0.317292 |
| *lincRNA:chr8:66501846-66512866_R* | -0.0709486 | 0.318368 |
| *lincRNA:chr15:50648133-50661733_R* | -0.0711755 | 0.31934 |
| *MAPKBP1* | -0.0713242 | 0.319826 |
| *XRCC1* | -0.0720261 | 0.32309 |
| *lincRNA:chr5:34044618-34053161_R* | -0.0736551 | 0.329583 |
| *PBX3* | -0.0737191 | 0.329792 |
| *SLC35F5* | -0.0738401 | 0.330521 |
| *LACTB* | -0.0755255 | 0.340104 |
| *DHRS12* | -0.0755876 | 0.340521 |
| *ZNF678* | -0.0759402 | 0.342153 |
| *LINC00341* | -0.0759765 | 0.342326 |
| *TIMM10* | -0.0770155 | 0.346632 |
| *SCLY* | -0.0772141 | 0.347222 |
| *SH3BGR* | -0.0778039 | 0.349861 |
| *XLOC_001537* | -0.0779036 | 0.350347 |
| *ITPR2* | -0.0780454 | 0.351076 |
| *DGCR11* | -0.0781473 | 0.351806 |
| *TATDN2* | -0.0784395 | 0.353715 |
| *lincRNA:chr1:88157912-88401162_R* | -0.0784495 | 0.35375 |
| *TMEM206* | -0.0787244 | 0.355139 |
| *FAHD1* | -0.0794732 | 0.358611 |
| *FIBIN* | -0.0798836 | 0.36066 |
| *lincRNA:chr5:43068217-43099440_R* | -0.0802436 | 0.362778 |
| *MRPL19* | -0.0808326 | 0.365938 |
| *CIB2* | -0.0811224 | 0.367361 |
| *GAR1* | -0.0811829 | 0.367604 |
| *lincRNA:chr4:83812701-83821676_R* | -0.0847444 | 0.378507 |
| *FERMT2* | -0.0850209 | 0.379201 |
| *MMACHC* | -0.0855752 | 0.381007 |
| *lincRNA:chr4:106271676-106285901_R* | -0.0858673 | 0.382083 |
| *lincRNA:chr1:247350513-247352101_R* | -0.0859893 | 0.382674 |
| *lincRNA:chr19:53702413-53709013_F* | -0.0870853 | 0.385035 |
| *CPVL* | -0.0873647 | 0.385521 |
| *AOAH* | -0.0874654 | 0.385833 |
| *TICAM1* | -0.0889948 | 0.390764 |
| *PPIA* | -0.0892887 | 0.392222 |
| *LRPPRC* | -0.0895371 | 0.39316 |
| *SERAC1* | -0.090233 | 0.395937 |
| *RGMB* | -0.090304 | 0.396146 |
| *LAMB1* | -0.0904418 | 0.396944 |
| *CCDC169* | -0.0916595 | 0.400833 |
| *SLC45A1* | -0.0927529 | 0.403194 |
| *PTPRD* | -0.0930904 | 0.404097 |
| *OSBPL11* | -0.0940634 | 0.407118 |
| *ARHGEF10* | -0.0947128 | 0.409549 |
| *DNM3* | -0.0948555 | 0.409965 |
| *lincRNA:chr4:6657715-6675623_R* | -0.0965225 | 0.415139 |
| *lincRNA:chr20:37049239-37049753_F* | -0.097351 | 0.417153 |
| *CENPW* | -0.0973797 | 0.417222 |
| *lincRNA:chr6:3708951-3719951_F* | -0.0975718 | 0.417778 |
| *TMEM63A* | -0.0977254 | 0.418021 |
| *ASRGL1* | -0.099995 | 0.423646 |
| *C1GALT1* | -0.101616 | 0.427326 |
| *CLEC16A* | -0.101974 | 0.427847 |
| *ANKRD32* | -0.102234 | 0.428299 |
| *LINC00271* | -0.102524 | 0.428819 |
| *lincRNA:chr11:107175165-107186315_R* | -0.10259 | 0.428924 |
| *WDR88* | -0.103227 | 0.430417 |
| *ATP5S* | -0.10374 | 0.432083 |
| *C5orf64* | -0.105356 | 0.435833 |
| *FRAS1* | -0.105375 | 0.435903 |
| *SLC36A1* | -0.106402 | 0.437639 |
| *FLJ33360* | -0.107007 | 0.438819 |
| *ADARB2* | -0.107371 | 0.439792 |
| *ATP1B3* | -0.107413 | 0.439861 |
| *E2F7* | -0.108894 | 0.445 |
| *TULP1* | -0.108905 | 0.445069 |
| *lincRNA:chr12:10727858-10733027_R* | -0.109325 | 0.446562 |
| *XLOC_011803* | -0.110634 | 0.450035 |
| *ZNF404* | -0.110819 | 0.450521 |
| *SLC8A3* | -0.110975 | 0.450868 |
| *ZFAT-AS1* | -0.111343 | 0.451632 |
| *CYB5R3* | -0.11225 | 0.454931 |
| *UBE2Z* | -0.112294 | 0.455069 |
| *lincRNA:chr17:17094547-17095960_F* | -0.112601 | 0.456319 |
| *ZNF264* | -0.113075 | 0.457639 |
| *lincRNA:chr20:37049265-37063981_F* | -0.114168 | 0.460729 |
| *lincRNA:chr8:590407-594400_R* | -0.116044 | 0.465 |
| *KCNG4* | -0.116854 | 0.467083 |
| *DLG4* | -0.117528 | 0.469167 |
| *CYBRD1* | -0.119517 | 0.47441 |
| *lincRNA:chr2:161109154-161115329_R* | -0.119775 | 0.474965 |
| *CCDC15* | -0.120004 | 0.475417 |
| *HIST1H1D* | -0.120127 | 0.47566 |
| *TSPAN15* | -0.120527 | 0.476424 |
| *HTATSF1P2* | -0.121018 | 0.477778 |
| *LRRC61* | -0.121376 | 0.478507 |
| *lincRNA:chr20:37049663-37063889_F* | -0.125812 | 0.488681 |
| *lincRNA:chr5:67089045-67101066_F* | -0.126332 | 0.489861 |
| *SEC13* | -0.126715 | 0.490764 |
| *TTC12* | -0.127532 | 0.493264 |
| *SMARCE1* | -0.128196 | 0.495799 |
| *lincRNA:chr1:234848577-234858502_R* | -0.128891 | 0.497049 |
| *PLA2G12A* | -0.130147 | 0.500938 |
| *lincRNA:chr2:152646148-152648600_R* | -0.130245 | 0.501076 |
| *HEATR3* | -0.130648 | 0.502153 |
| *lincRNA:chr5:44828220-44828690_F* | -0.130712 | 0.502361 |
| *XPNPEP3* | -0.132088 | 0.504965 |
| *TBCE* | -0.132101 | 0.504965 |
| *RBM26-AS1* | -0.133175 | 0.506701 |
| *lincRNA:chr6:86387201-86388404_R* | -0.134312 | 0.508958 |
| *SUPT7L* | -0.134427 | 0.509167 |
| *KCNQ2* | -0.134785 | 0.509757 |
| *MED10* | -0.137204 | 0.516319 |
| *lincRNA:chr2:123722255-124760730_R* | -0.137855 | 0.517431 |
| *DHRS11* | -0.139022 | 0.518507 |
| *lincRNA:chr12:28212358-28218083_R* | -0.139845 | 0.519792 |
| *CHRNA5* | -0.140455 | 0.521042 |
| *lincRNA:chr7:30614038-30616352_R* | -0.140908 | 0.521701 |
| *DECR2* | -0.141342 | 0.522396 |
| *MCF2L* | -0.141638 | 0.522847 |
| *lincRNA:chr2:62367371-62385846_F* | -0.142621 | 0.523681 |
| *SFTA1P* | -0.144038 | 0.52566 |
| *CAND1* | -0.144191 | 0.525903 |
| *AMACR* | -0.144625 | 0.526563 |
| *SNX19* | -0.147342 | 0.529549 |
| *FGL2* | -0.148539 | 0.531007 |
| *ZNF720* | -0.149393 | 0.531736 |
| *UACA* | -0.151846 | 0.534653 |
| *TREX1* | -0.155016 | 0.538958 |
| *ZNF441* | -0.155923 | 0.540278 |
| *TMEM8B* | -0.157913 | 0.542847 |
| *lincRNA:chr10:92927295-92961870_R* | -0.158868 | 0.543854 |
| *SETD3* | -0.160439 | 0.545451 |
| *IL23A* | -0.160628 | 0.545729 |
| *PRKXP1* | -0.160871 | 0.546076 |
| *CSGALNACT2* | -0.164473 | 0.551771 |
| *FLJ42393* | -0.164971 | 0.552535 |
| *SLC25A37* | -0.165082 | 0.552778 |
| *ABCC3* | -0.165301 | 0.553056 |
| *lincRNA:chr1:247305102-247313777_R* | -0.165764 | 0.554167 |
| *RAB3B* | -0.166299 | 0.55559 |
| *SUMF1* | -0.166313 | 0.555625 |
| *UBE2U* | -0.166919 | 0.557431 |
| *ATG10* | -0.168022 | 0.558681 |
| *BLOC1S5* | -0.169596 | 0.560347 |
| *SNRNP27* | -0.170694 | 0.562674 |
| *TMEM244* | -0.171479 | 0.563715 |
| *lincRNA:chr6:86386853-86387908_R* | -0.172516 | 0.565139 |
| *GOSR2* | -0.17562 | 0.568507 |
| *MTHFSD* | -0.178689 | 0.572986 |
| *lincRNA:chr5:44826637-44828692_F* | -0.18063 | 0.575729 |
| *lincRNA:chr1:101548009-101552817_F* | -0.181193 | 0.576562 |
| *BREA2* | -0.181351 | 0.576806 |
| *lincRNA:chr8:103939574-104019049_F* | -0.182315 | 0.577674 |
| *lincRNA:chr4:8357036-8359103_R* | -0.182624 | 0.578125 |
| *CHRM5* | -0.184303 | 0.580278 |
| *lincRNA:chr7:30589933-30601268_R* | -0.186349 | 0.58316 |
| *XLOC_009313* | -0.189501 | 0.586701 |
| *lincRNA:chr6:86387687-86387908_R* | -0.19022 | 0.58809 |
| *LDHC* | -0.191152 | 0.589132 |
| *lincRNA:chr6:86386801-86387745_F* | -0.19219 | 0.590208 |
| *HS1BP3* | -0.192808 | 0.590729 |
| *RSRC1* | -0.195934 | 0.594757 |
| *STMN4* | -0.196025 | 0.594931 |
| *RHCE* | -0.19843 | 0.598854 |
| *PPIL6* | -0.198509 | 0.599063 |
| *ARL16* | -0.202669 | 0.6025 |
| *RPUSD2* | -0.204977 | 0.604236 |
| *TIE1* | -0.205774 | 0.604965 |
| *MEGF11* | -0.20637 | 0.605556 |
| *MYO5A* | -0.20669 | 0.605868 |
| *PBLD* | -0.20963 | 0.608194 |
| *lincRNA:chr6:132278082-132290382_F* | -0.210184 | 0.608472 |
| *ZNF568* | -0.211126 | 0.609236 |
| *lincRNA:chr6:28083684-28084323_R* | -0.213554 | 0.611319 |
| *ABCA6* | -0.215397 | 0.612986 |
| *NEK5* | -0.218773 | 0.616389 |
| *LSM7* | -0.219609 | 0.617569 |
| *GHRLOS* | -0.221649 | 0.620139 |
| *SCRG1* | -0.222965 | 0.621528 |
| *CYP4F3* | -0.223513 | 0.622153 |
| *lincRNA:chr13:21897375-21908850_F* | -0.22409 | 0.622882 |
| *GNLY* | -0.227749 | 0.626424 |
| *DDR2* | -0.230734 | 0.628958 |
| *lincRNA:chr15:52216592-52223505_F* | -0.231612 | 0.629653 |
| *SERPINB9* | -0.233263 | 0.631597 |
| *EFCAB1* | -0.233771 | 0.632118 |
| *ANKRD12* | -0.235043 | 0.633125 |
| *PHLDB3* | -0.236277 | 0.634306 |
| *KIAA1683* | -0.237752 | 0.635833 |
| *RPA1* | -0.238631 | 0.636597 |
| *lincRNA:chr6:86386796-86387718_R* | -0.242009 | 0.640104 |
| *lincRNA:chr6:86387140-86387958_R* | -0.246363 | 0.645174 |
| *lincRNA:chr7:30587974-30601744_R* | -0.246911 | 0.646146 |
| *lincRNA:chr6:86386863-86387906_R* | -0.250953 | 0.651181 |
| *MS4A14* | -0.251778 | 0.652813 |
| *DMXL2* | -0.251894 | 0.653056 |
| *lincRNA:chr4:8357101-8357609_R* | -0.253515 | 0.655868 |
| *SHPK* | -0.255674 | 0.658819 |
| *PPM1H* | -0.257028 | 0.660694 |
| *lincRNA:chr22:24251300-24274300_R* | -0.258389 | 0.662778 |
| *TIMM13* | -0.259968 | 0.664722 |
| *FAS* | -0.260348 | 0.664965 |
| *Sep-12* | -0.262599 | 0.6675 |
| *KRTAP4-2* | -0.265 | 0.670035 |
| *PRPH2* | -0.265312 | 0.670486 |
| *lincRNA:chr6:86387186-86387977_R* | -0.269452 | 0.674271 |
| *MSH3* | -0.273032 | 0.677222 |
| *NR2F6* | -0.273593 | 0.677604 |
| *PIGZ* | -0.274176 | 0.678472 |
| *MAEL* | -0.274287 | 0.678785 |
| *HLA-DRB1* | -0.274876 | 0.679688 |
| *SNX13* | -0.274996 | 0.679826 |
| *NKAIN1* | -0.278228 | 0.683333 |
| *CCDC7* | -0.279949 | 0.685 |
| *lincRNA:chr6:86387517-86388451_R* | -0.280381 | 0.685382 |
| *DHFR* | -0.280465 | 0.685451 |
| *METTL21B* | -0.280596 | 0.68559 |
| *lincRNA:chr6:86386850-86387907_F* | -0.280863 | 0.686042 |
| *lincRNA:chr18:72258320-72282445_R* | -0.281243 | 0.686667 |
| *CCDC137* | -0.281362 | 0.686806 |
| *ESRRG* | -0.284156 | 0.689583 |
| *ITGB7* | -0.285639 | 0.691632 |
| *AGMAT* | -0.286695 | 0.693785 |
| *TMEM99* | -0.286845 | 0.694132 |
| *RPS14* | -0.29218 | 0.700174 |
| *lincRNA:chr13:21906119-21906571_F* | -0.293015 | 0.700764 |
| *ZNF528* | -0.294687 | 0.702326 |
| *AKAP10* | -0.295588 | 0.703472 |
| *SMUG1* | -0.297297 | 0.705903 |
| *IFITM10* | -0.298475 | 0.707326 |
| *lincRNA:chr6:86386801-86387887_F* | -0.299393 | 0.708333 |
| *NUMBL* | -0.299588 | 0.708507 |
| *lincRNA:chr6:86386847-86387869_R* | -0.301926 | 0.710938 |
| *lincRNA:chr4:15685948-15688226_F* | -0.30966 | 0.718403 |
| *CRYGB* | -0.310328 | 0.718993 |
| *lincRNA:chr2:75147817-75175517_R* | -0.310564 | 0.719097 |
| *ZNF593* | -0.313929 | 0.722083 |
| *lincRNA:chr6:86386876-86387701_R* | -0.315206 | 0.722847 |
| *MXI1* | -0.315388 | 0.722917 |
| *UGDH* | -0.316075 | 0.723403 |
| *lincRNA:chr6:86386844-86387863_R* | -0.319231 | 0.725799 |
| *lincRNA:chr6:86386841-86387899_R* | -0.320593 | 0.726736 |
| *PPIL3* | -0.321687 | 0.727951 |
| *lincRNA:chr6:86386840-86387907_R* | -0.322164 | 0.72875 |
| *lincRNA:chr6:86386083-86388435_F* | -0.325242 | 0.731319 |
| *SNHG5* | -0.326862 | 0.733368 |
| *EIF2AK1* | -0.327567 | 0.734618 |
| *ALDH8A1* | -0.327854 | 0.735035 |
| *lincRNA:chr6:86387160-86387908_R* | -0.332457 | 0.740139 |
| *IRF5* | -0.335269 | 0.742465 |
| *CBLN3* | -0.335845 | 0.742882 |
| *ABHD11* | -0.336994 | 0.743715 |
| *SURF1* | -0.33804 | 0.744375 |
| *WDSUB1* | -0.338753 | 0.745069 |
| *CLEC4GP1* | -0.343107 | 0.748368 |
| *lincRNA:chr7:54850800-54872662_F* | -0.347208 | 0.750694 |
| *SYT1* | -0.347485 | 0.750938 |
| *ERAP2* | -0.349013 | 0.751597 |
| *FDXR* | -0.351957 | 0.753472 |
| *lincRNA:chr7:105564174-105564567_F* | -0.352209 | 0.75375 |
| *lincRNA:chr6:86386801-86387731_F* | -0.354639 | 0.755104 |
| *ACER3* | -0.362099 | 0.757396 |
| *lincRNA:chr6:86386825-86387901_R* | -0.364644 | 0.758924 |
| *SSPO* | -0.365956 | 0.759271 |
| *lincRNA:chr6:86386831-86387899_R* | -0.376541 | 0.764201 |
| *lincRNA:chr6:86386799-86387711_F* | -0.384813 | 0.768472 |
| *HYI* | -0.385244 | 0.76875 |
| *MRPL42P5* | -0.396132 | 0.773507 |
| *lincRNA:chr3:128147010-128171232_R* | -0.403998 | 0.777882 |
| *FLJ46906* | -0.407819 | 0.779444 |
| *CHURC1* | -0.409016 | 0.779931 |
| *CCDC154* | -0.409247 | 0.780035 |
| *lincRNA:chr8:110235749-110247024_R* | -0.414279 | 0.784757 |
| *ZNFX1* | -0.418257 | 0.786319 |
| *lincRNA:chr6:86386813-86387908_F* | -0.422043 | 0.788333 |
| *CD47* | -0.422712 | 0.788438 |
| *lincRNA:chr6:86386802-86388443_R* | -0.42463 | 0.789028 |
| *RPL22L1* | -0.446116 | 0.797639 |
| *XLOC_013825* | -0.446852 | 0.797813 |
| *C5orf55* | -0.446937 | 0.797813 |
| *CARD8* | -0.455438 | 0.801319 |
| *ORMDL1* | -0.458783 | 0.802465 |
| *lincRNA:chr14:48681275-48724375_R* | -0.463758 | 0.804132 |
| *PHOSPHO2* | -0.465415 | 0.804653 |
| *NQO2* | -0.466074 | 0.805 |
| *lincRNA:chr20:1316227-1317552_R* | -0.466628 | 0.805174 |
| *GSTT2B* | -0.468685 | 0.806424 |
| *OGFOD1* | -0.470601 | 0.807292 |
| *AQP4-AS1* | -0.48646 | 0.812986 |
| *ZNF124* | -0.488503 | 0.813993 |
| *EIF2A* | -0.498432 | 0.816806 |
| *GREB1* | -0.503943 | 0.819167 |
| *SLC28A2* | -0.505737 | 0.820035 |
| *lincRNA:chr18:5238975-5246369_F* | -0.51418 | 0.824201 |
| *SNX31* | -0.518843 | 0.825486 |
| *BOD1L1* | -0.53031 | 0.829444 |
| *GNMT* | -0.536632 | 0.831632 |
| *lincRNA:chr9:2530903-2539456_R* | -0.547466 | 0.83434 |
| *NOL12* | -0.557019 | 0.837708 |
| *MRVI1* | -0.564131 | 0.840938 |
| *DBC1* | -0.566754 | 0.841875 |
| *GLYR1* | -0.569382 | 0.843437 |
| *LPIN1* | -0.570486 | 0.843681 |
| *GPN1* | -0.574281 | 0.844861 |
| *LYPD5* | -0.574428 | 0.844896 |
| *ZNF252P* | -0.584944 | 0.848785 |
| *C2orf74* | -0.586936 | 0.849479 |
| *TMEM80* | -0.59284 | 0.851562 |
| *FBXL17* | -0.601674 | 0.855382 |
| *MEIG1* | -0.601787 | 0.855417 |
| *C11orf21* | -0.611344 | 0.858854 |
| *XRRA1* | -0.613232 | 0.85934 |
| *IVD* | -0.617036 | 0.860903 |
| *EFCAB2* | -0.618962 | 0.862083 |
| *CWF19L1* | -0.619746 | 0.862569 |
| *QRSL1* | -0.626363 | 0.865799 |
| *SETD9* | -0.62665 | 0.865938 |
| *WFIKKN1* | -0.632639 | 0.87 |
| *DDT* | -0.642688 | 0.876597 |
| *lincRNA:chr20:37049312-37059741_F* | -0.650634 | 0.878611 |
| *C7orf29* | -0.666098 | 0.883646 |
| *GFM1* | -0.680889 | 0.886979 |
| *WARS2* | -0.689801 | 0.888924 |
| *FLVCR1-AS1* | -0.696082 | 0.890868 |
| *SNRPB2* | -0.703947 | 0.894479 |
| *lincRNA:chr14:95873047-95878997_F* | -0.703949 | 0.894479 |
| *SPSB2* | -0.705999 | 0.895556 |
| *FAM118A* | -0.712533 | 0.898819 |
| *ANKRD42* | -0.713006 | 0.899097 |
| *ARPC5* | -0.713041 | 0.899097 |
| *LINC00476* | -0.720043 | 0.902396 |
| *UNC13C* | -0.725225 | 0.904132 |
| *KIF3A* | -0.735498 | 0.908125 |
| *lincRNA:chr16:88737774-88742899_F* | -0.736792 | 0.908681 |
| *RABEP1* | -0.740019 | 0.909549 |
| *C16orf88* | -0.742306 | 0.910104 |
| *ACCS* | -0.743702 | 0.910556 |
| *DNAJC15* | -0.743709 | 0.910556 |
| *SLC25A51* | -0.745521 | 0.911285 |
| *RMDN1* | -0.756377 | 0.915104 |
| *ULK4* | -0.763224 | 0.916424 |
| *GTPBP5* | -0.767678 | 0.917639 |
| *FAM200B* | -0.769349 | 0.918125 |
| *PNLDC1* | -0.769841 | 0.918299 |
| *CCDC174* | -0.772946 | 0.919271 |
| *lincRNA:chr4:68586468-68587852_F* | -0.778946 | 0.920868 |
| *SLC4A7* | -0.78012 | 0.921285 |
| *ABCA9* | -0.808848 | 0.932049 |
| *lincRNA:chr20:37037086-37082161_R* | -0.809847 | 0.932257 |
| *THAP7-AS1* | -0.81344 | 0.933611 |
| *lincRNA:chr6:86386806-86387736_F* | -0.813788 | 0.933715 |
| *SOHLH2* | -0.824527 | 0.937743 |
| *CCDC73* | -0.824703 | 0.937847 |
| *ZNF667* | -0.828314 | 0.939375 |
| *LOC100505679* | -0.828477 | 0.93941 |
| *EFHB* | -0.828966 | 0.939583 |
| *lincRNA:chr7:105551484-105564591_F* | -0.831703 | 0.94125 |
| *lincRNA:chr4:68586647-68587436_F* | -0.850752 | 0.949063 |
| *FTSJ3* | -0.851743 | 0.949826 |
| *NS3BP* | -0.861295 | 0.953819 |
| *PCDHB7* | -0.861968 | 0.953993 |
| *lincRNA:chr4:15657202-15695627_F* | -0.86645 | 0.955 |
| *lincRNA:chr3:156894106-156971481_F* | -0.868727 | 0.955694 |
| *SIPA1L1* | -0.870442 | 0.956076 |
| *MTRR* | -0.871889 | 0.956528 |
| *DDTL* | -0.876203 | 0.957465 |
| *GBP3* | -0.876698 | 0.957639 |
| *SFXN4* | -0.889471 | 0.961632 |
| *NUPL2* | -0.89027 | 0.96184 |
| *lincRNA:chr4:68586523-68587641_F* | -0.894429 | 0.963646 |
| *TMEM14B* | -0.904076 | 0.966285 |
| *lincRNA:chr15:50656197-50659350_F* | -0.906945 | 0.967778 |
| *lincRNA:chr15:50648133-50663574_F* | -0.927234 | 0.971806 |
| *lincRNA:chr15:50653887-50654969_F* | -0.929628 | 0.972326 |
| *MRPL43* | -0.929973 | 0.972361 |
| *SCAPER* | -0.935077 | 0.973368 |
| *lincRNA:chr6:155203224-155205268_F* | -0.93949 | 0.974479 |
| *ACOXL* | -0.940742 | 0.974826 |
| *SIGLEC11* | -0.942672 | 0.975556 |
| *TMPRSS5* | -0.962798 | 0.979688 |
| *THNSL2* | -0.97147 | 0.981701 |
| *WBSCR27* | -0.971839 | 0.981736 |
| *lincRNA:chr11:47623024-47633724_R* | -0.976388 | 0.982917 |
| *PEX6* | -0.979361 | 0.983715 |
| *TCFL5* | -0.989508 | 0.985903 |
| *YTHDC1* | -0.992838 | 0.986944 |
| *lincRNA:chr2:75158491-75160151_R* | -1.00362 | 0.991076 |
| *ELP5* | -1.01392 | 0.995764 |
| *UBA52* | -1.01416 | 0.995799 |

# 3. Supplemental Results: Supplemental Figures

Figure S1 Enrichment of significant SNPs from three eQTL studies (epilepsy-eQTL, GTEx and Braineac) within SNPs from the FS GWAS. Epi-eQTL = epilepsy-eQTL; p-value notations as in the following example: 1e^-6^ = 1x10^-6^.


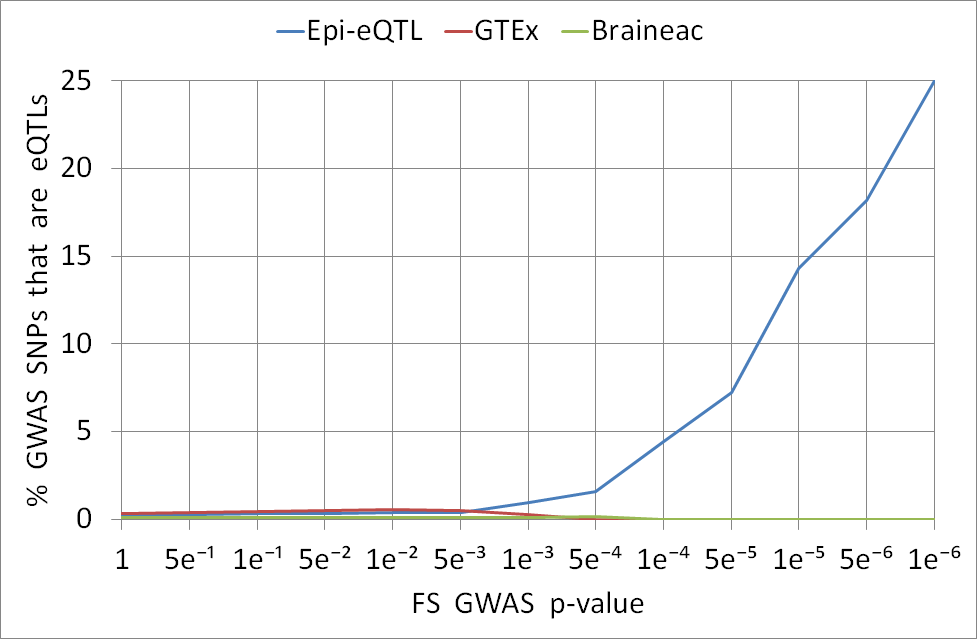


# 4. Full eQTL results

Full eQTL results are provided as a supplemental data file (eQTL_results.bz2). In order to keep the file size to a minimum, only essential data is provided in a bz2 compressed file. The file can be uncompressed by running bunzip2 eQTL_results.bz2 from a unix command-line. The file contains SNP rs identifier, chromosome, base-position (build GRCh37/hg19), allele 1, allele 2, probe identifier, gene symbol, Entrez gene identifier, beta effect size estimate, t-statistic, p-value and false discovery rate. Further information about probes, for example sequences and coordinates, can be obtained individually or in batch using these freely available web resources: Agilent’s eArray (<https://earray.chem.agilent.com/earray/>) or Ensembl ([www.ensembl.org/](http://www.ensembl.org/)).

# 5. References

1 Kim, Y., Xia, K., Tao, R., Giusti-Rodriguez, P., Vladimirov, V., van den Oord, E. and Sullivan, P.F. (2014) A meta-analysis of gene expression quantitative trait loci in brain. *Translational psychiatry*, **4**, e459.

2 Li, L., Kabesch, M., Bouzigon, E., Demenais, F., Farrall, M., Moffatt, M.F., Lin, X. and Liang, L. (2013) Using eQTL weights to improve power for genome-wide association studies: a genetic study of childhood asthma. *Frontiers in genetics*, **4**, 103.

3 Chang, C.C., Chow, C.C., Tellier, L.C., Vattikuti, S., Purcell, S.M. and Lee, J.J. (2015) Second-generation PLINK: rising to the challenge of larger and richer datasets. *GigaScience*, **4**, 7.

4 Auton, A., Brooks, L.D., Durbin, R.M., Garrison, E.P., Kang, H.M., Korbel, J.O., Marchini, J.L., McCarthy, S., McVean, G.A. and Abecasis, G.R. (2015) A global reference for human genetic variation. *Nature*, **526**, 68-74.

5 Mirza, N., Appleton, R., Burn, S., Carr, D., Crooks, D., du Plessis, D., Duncan, R., Farah, J.O., Josan, V., Miyajima, F. *et al.* (2015) Identifying the biological pathways underlying human focal epilepsy: from complexity to coherence to centrality. *Human molecular genetics*, **24**, 4306-4316.

6 (2014) Genetic determinants of common epilepsies: a meta-analysis of genome-wide association studies. *Lancet neurology*, **13**, 893-903.

7 He, X., Fuller, C.K., Song, Y., Meng, Q., Zhang, B., Yang, X. and Li, H. (2013) Sherlock: detecting gene-disease associations by matching patterns of expression QTL and GWAS. *American journal of human genetics*, **92**, 667-680.
